# scFv-Anti-LDL(-)-Metal-Complex Multi-Wall Functionalized-Nanocapsules as a Promising Tool for the Prevention of Atherosclerosis Progression

**DOI:** 10.3389/fmed.2021.652137

**Published:** 2021-04-20

**Authors:** Marcela Frota Cavalcante, Márcia Duarte Adorne, Walter Miguel Turato, Marina Kemmerer, Mayara Klimuk Uchiyama, Ana Carolina Cavazzin Asbahr, Aline de Cristo Soares Alves, Sandra Helena Poliselli Farsky, Carine Drewes, Marina Cecília Spatti, Soraya Megumi Kazuma, Marcel Boss, Silvia Stanisçuaski Guterres, Koiti Araki, Bernhard Brüne, Dmitry Namgaladze, Adriana Raffin Pohlmann, Dulcineia Saes Parra Abdalla

**Affiliations:** ^1^Department of Clinical and Toxicological Analysis, Faculty of Pharmaceutical Sciences, University of São Paulo, São Paulo, Brazil; ^2^Department of Organic Chemistry, Chemistry Institute, Federal University of Rio Grande do Sul, Porto Alegre, Brazil; ^3^Faculty of Medicine, Institute of Biochemistry I, Goethe-University Frankfurt, Frankfurt, Germany; ^4^Department of Fundamental Chemistry, Institute of Chemistry, University of São Paulo, São Paulo, Brazil; ^5^Department of Production and Control of Medicines, Faculty of Pharmacy, Federal University of Rio Grande do Sul, Porto Alegre, Brazil

**Keywords:** atherosclerosis, nanocapsules, electronegative LDL, macrophage, single chain fragment variable, nanoformulation

## Abstract

Atherosclerosis can be originated from the accumulation of modified cholesterol-rich lipoproteins in the arterial wall. The electronegative LDL, LDL(-), plays an important role in the pathogenesis of atherosclerosis once this cholesterol-rich lipoprotein can be internalized by macrophages, contributing to the formation of foam cells, and provoking an immune-inflammatory response. Herein, we engineered a nanoformulation containing highly pure surface-functionalized nanocapsules using a single-chain fragment variable (scFv) reactive to LDL(-) as a ligand and assessed whether it can affect the LDL(-) uptake by primary macrophages and the progression of atherosclerotic lesions in *Ldlr*^−/−^ mice. The engineered and optimized scFv-anti-LDL(-)-MCMN-Zn nanoformulation is internalized by human and murine macrophages *in vitro* by different endocytosis mechanisms. Moreover, macrophages exhibited lower LDL(-) uptake and reduced mRNA and protein levels of *IL1B* and MCP1 induced by LDL(-) when treated with this new nanoformulation. In a mouse model of atherosclerosis employing *Ldlr*^−/−^ mice, intravenous administration of scFv-anti-LDL(-)-MCMN-Zn nanoformulation inhibited atherosclerosis progression without affecting vascular permeability or inducing leukocytes-endothelium interactions. Together, these findings suggest that a scFv-anti-LDL(-)-MCMN-Zn nanoformulation holds promise to be used in future preventive and therapeutic strategies for atherosclerosis.

## Introduction

In the last years, nanoparticles have been widely proposed for medical applications (1) either for diagnosis, therapy or molecular imaging (2). This great acceptance is related to their properties presented by nanoparticles such as their versatility of formulation, colloidal size, biocompatibility and sustained-release properties (3). This set of characteristics has also encouraged the use of nanoparticles as drug carriers, allowing the delivery of drugs, peptides, antibodies and recombinant proteins at a specific rate and at a specific sites (4). The use of synthetic polymer nanoparticles and nanoparticle-protein conjugates, or functionalized nanoparticles, as nanocarriers improves the effectiveness of potential biodrugs, providing promising advantages, such as improved stability, a precise control of their pharmacokinetics and bioavailability and increased residence time in the body (5). Moreover, nanoparticles have greater interaction with cells due to their larger contact surface (6) and can facilitate delivery across cell membranes and organ barriers (e.g., blood-brain barrier) (7) besides enabling the safe delivery of toxic therapeutic compounds, protecting non-target tissues and cells from possible side effects (5).

Although Nanomedicine's applications were initially focused on Oncology, a substantial number of studies have been conducted in the last decade in cardiovascular diseases, mainly in atherosclerosis (8). Atherosclerosis is a complex and chronic, lipid and inflammation-driven disease characterized by the accumulation of modified cholesterol-rich lipoproteins in the arterial wall of large- and medium-sized arteries (9). Briefly, the accumulation of modified low-density lipoprotein (LDL) particles, such as the electronegative LDL [LDL(-)], can trigger the activation of the endothelium activation and recruitment of monocytes and T cells into the arterial intima. The infiltrated immune cells, particularly monocyte-derived macrophages, uptake modified LDL particles via scavenger receptors-mediated endocytosis leading to the formation of foam cells and release of pro-inflammatory cytokines (10). Besides pro-inflammatory properties, LDL (-) can induce angiogenesis contributing to plaque instability (11), making this lipoprotein subfraction an excellent candidate for specific targeting therapy.

In this study, we aimed to optimize the synthesis of lecithin-chitosan-coated lipid-core nanocapsules having at the surface, as ligand, a scFv (single chain fragment variable) reactive to LDL(-) as a ligand, and to assess its effects on the uptake by human and murine primary macrophages. Moreover, we investigated whether this scFv-anti-LDL(-)-MCMN-Zn nanoformulation inhibits the progression of atherosclerosis in *Ldlr*^−/−^ mice, evaluating its promising use in drug delivery and as a targeting approaches to prevention and treat the progression of atherosclerosis.

## Materials and Methods

### Human and Animal Studies

The study was approved by the Human Research Ethics Committee for the use of human blood (n. 114/2010), and by the Animal Research Ethics Committee, for the use of low-density lipoprotein receptor-deficient mice (*Ldlr*^−/−^,C57BL/6J homozygous background) (n. 392/2013), both of the Faculty of Pharmaceutical Sciences from the University of São Paulo, in agreement with the Brazilian College for Animal Experimentation guidelines.

### Materials

Poly(e-caprolactone) (PCL) (#440752; a,w-dihydroxy functional polymer, M_n_ 10,000 g/mol, M_w_ 14,000 g/mol), sorbitan monostearate (Span 60®, #S7010), chitosan (#48869; M_w_ 50,000–190,000 g/mol, 75-85% deacetylated), zinc acetate (#383317), and cholic acid (#135240) were acquired from Sigma-Aldrich (Saint-Quentin-Fallavier, FR). Capric-caprylic triglyceride (CAS#73398-61-5) and polysorbate 80 (Tween 80®, #P1754) were delivered by Delaware (Porto Alegre, BR). Soybean lecithin (Lipoid S75®, #776132-15/301) was obtained from Lipoid (Ludwigshafen, DE). Acetone and ethanol (analytical grade) were used.

### Synthesis Optimization of Chitosan-Lecithin-Coated Lipid-Core Nanocapsules

#### Lecithin-Lipid-Core Nanocapsules

A pre-formulation study to determine the optimized concentration of lecithin in the lipid-core nanocapsules (LNC) was conducted. Formulations were named considering the lecithin concentration (Supplementary Material).

#### Chitosan Coating of Lecithin-Lipid-Core Nanocapsules

A pre-formulation study to determine the optimized concentration of chitosan to coat the lecithin-lipid-core nanocapsules was conducted. Nine different formulations were prepared (Supplementary Material) by varying the chitosan concentration (0.5 to 1.4 mg/mL). For the biological evaluations, the best formulation (${\text{LNC}}_{0.7}^{+}$) was prepared using 1% acetic acid.

### Obtention of scFv-anti-LDL(-)

The scFv-anti-LDL(-) was expressed in the yeast *Pichia pastoris* (SMD1168 strain—#C17500, ThermoFisher Scientific, MA, USA), as previously reported (12) (Supplementary Material).

### Surface Functionalization to Produce the scFv-anti-LDL(-)-MCMN-Zn Nanoformulation

${\text{LNC}}_{0.7}^{+}$ was added of zinc acetate solution, composing the MCMN (Metal Complex Multiwalled Nanocapsules) complex, and, after 1 min, of scFv-anti-LDL(-) aqueous solution (Supplementary Material).

### Isolation of LDL(-)

The isolation of LDL(-) was performed by sequential flotation ultracentrifugation according to Faulin et al. (13) (Supplementary Material).

### Cell Isolation and Culture Conditions

Human monocytes were isolated from peripheral blood (14) using Ficoll gradient (#17-829E, Lonza, MO, USA), Leucosep^TM^ tubes (#227290, Greiner Bio-One, NC, USA) and CD14 microbeads selection system (#130-050-201, Miltenyi Biotec, CA, USA) and differentiated (Supplementary Material) into macrophages with human recombinant macrophage colony-stimulating factor (M-CSF, #78057, StemCell Technologies, WA, USA) as described in the Supplementary Material. To obtain murine primary macrophages (Supplementary Material), bone marrow cells were isolated from femur and tibia of C57BL/6J *Ldlr*^−/−^ mice and differentiated (Supplementary Material) in the presence of growth factors as previously described (15). For treatments, both macrophages were cultured in RPMI 1640 medium containing 2 mM L-glutamine, 2 g/L sodium bicarbonate (#S5761, Sigma-Aldrich, São Paulo, Brazil), and a mix of 100 μg/mL streptomycin and 100 U/mL penicillin (#15070-063, Gibco®, MA, USA) and 10% fetal bovine serum. The concentration of FBS was reduced to 1% for 16 h before the treatments. For flow cytometry assays, 2.5 × 10^5^ murine or human primary macrophages were seeded in 12-well plates, exposed to the treatments, detached with sequential pipetting and resuspended in PBS for analysis. For confocal microscopy, 4-well CELLview^TM^ glass-bottom dishes (#627975, Greiner Bio-One, Frickenhausen, DE) were used for the culture of 2.5 × 10^5^ murine or human primary macrophages. These cells were treated and, after 3 h, fixed with 10% neutral buffered formaldehyde for 15 min. The fluorophore DAPI (4′,6-Diamidino-2-Phenylindole Dihydrochloride, #D1306, Gibco®, NY, USA) was added to stain the cells nuclei. All experiments included a control group that did not receive any treatment.

### Cellular Uptake of scFv-anti-LDL(-)-MCMN-Zn Nanoformulation

#### Flow Cytometry and Confocal Microscopy

The internalization of the scFv-anti-LDL(-)-MCMN-Zn nanoformulation by murine and human macrophages was evaluated by flow cytometry and confocal microscopy. The fluorophore Rhodamine B was chemically bound to PCL and the PCL-RhoB conjugate was incorporated into the nanocapsule structure during the first step of its synthesis using a blend PCL-RhoB/PCL (1:10, w/w). Cells were incubated with 10^4^ particles/mL scFv-anti-LDL(-)-MCMN-Zn nanoformulation (containing 6.25 μg of scFv protein/mL of nanoformulation) for 3 h at 37°C and then evaluated. For flow cytometry assays, all samples were analyzed with a FACSCanto flow cytometer (BD Biosciences, NJ, USA) and the data analysis of 10,000 events, for each experimental condition, was performed using Flow Jo software (version 9.5.1, Tree Star Inc, OR, USA). For confocal microscopy analysis, images were obtained with a Zeiss LSM 510 Meta confocal microscope with a HAL 100 illuminator (Carl Zeiss Microscopy, Cambridge, UK).

#### Fluorescence and Hyperspectral Microscopy by CytoViva®

The fluorescence and enhanced darkfield hyperspectral microscopy were performed to evaluate the internalization of the nanoformulation by the macrophages of both species using a CytoViva® Ultra Resolution Imaging System (CytoViva, Inc., AL, USA) mounted on an Olympus BX51 microscope (×1,500 magnification; Olympus, Tokyo, JP). Briefly, 2.0 × 10^5^ cells/well were seeded in extra clean dust-free Nexterion® Glass D coverslips (#D263T, Schott, NY, USA) present in 6-well plates and incubated with 10^4^ particles/mL of non-targeting Phe-MCMN-Zn nanoparticles or scFv-anti-LDL(-)-MCMN-Zn nanoformulation (containing 6.25 μg of scFv protein/mL of nanoformulation), containing the fluorophore Rhodamine B (PCL-RhoB), for 3 h at 37°C. After the treatment, coverslips were carefully placed on slides containing 10 μL of PBS and imaged. Images were acquired with a Dage XL CCD digital camera and Image Processing Software (DAGE®, Japan). Data were collected from multiple areas using the Specim V10E CCD spectral camera, and the respective average spectrum for each sample was determined using the CytoViva® customized ENVI Hyperspectral Image Analysis software.

### Endocytosis Study

The mechanisms involved in the internalization of the scFv-anti-LDL(-)-MCMN-Zn nanoformulation by murine and human macrophages were examined by flow cytometry. Cells were treated with 10^4^ particles/mL of scFv-anti-LDL(-)-MCMN-Zn nanoformulation (containing 6.25 μg of scFv protein/mL of nanoformulation) for 3 h at 37°C, after 1 h pretreatment with the following endocytosis inhibitors: 50 μM Chloroquine, 40 μM Cytochalasin D, 50 μM EIPA [5-(N-Ethyl-N-isopropyl) amiloride], 80 μM Dynasore and 1 μM Brefeldin A for inhibition of endocytosis, phagocytosis, macropinocytosis, dynamin-dependent endocytosis and intracellular protein transport, respectively.

### Cellular Uptake of LDL(-)

The effect of the scFv-anti-LDL(-)-MCMN-Zn nanoformulation on the uptake of LDL(-) by both species of macrophages was evaluated by flow cytometry. Cells were treated with 10^4^ particles/mL of scFv-anti-LDL(-)-MCMN-Zn nanoformulation (containing 6.25 ug of scFv protein/ml of nanoformulation) and LDL(-) solution (containing 37.5 μg of LDL(-) protein/mL) individually or combined for 3 h at 37°C. The LDL(-) was labeled with the fluorophore DiI(C_18_) (1,1′-Dioctadecyl-3,3,3′,3′-Tetramethylindocarbocyanine Perchlorate, #D282, Life Technologies, USA) (16).

### Gene Expression Analysis by qRT-PCR

Levels of mRNA were determined by quantitative RT-PCR on a PCR 7,500 Fast Real-time PCR System (Applied Biosystems, MA, USA). Cells were treated with 10^4^ particles/mL of scFv-anti-LDL(-)-MCMN-Zn nanoformulation (containing 6.25 ug of scFv protein/mL of nanoformulation) and LDL(-) (containing 37.5 μg of LDL(-) protein/mL) individually or combined for 24 h at 37°C. Primers for *IL1B* and *MCP1* were used (Supplementary Table 1). Expression of each target gene was normalized with *GAPDH* for human and *Rpl13a* and for murine macrophages and calculated by the 2^(−ΔΔCt)^ method (17).

### Expression of IL-1β by Cytometric Beads Array (CBA)

Protein levels of IL-1β, CBA assays were assessed by flow cytometry, using the Human IL-1β Flex Set Kit (#558279, BD Biosciences, CA, USA) or the Mouse IL-1β Enhanced Sensitivity Flex Set Kit (#562278, BD Biosciences, CA, USA). The supernatant of 1 × 10^6^ cells after 48 h of treatment at 37°C as described before, and the samples were prepared following manufacturer's instructions.

### Safety Evaluation of Nanoformulation in C57BL/6J *Ldlr*^–/–^ Mice

The safety of the intravenous administration of the nanoformulation was investigated by intravital microscopy (18, 19). The effects of the nanoformulation on microcirculation and vascular permeability were evaluated in 12-weeks-old male *Ldlr*^−/−^ mice previously fed for 8 weeks either a standard chow (Rhoster®, São Paulo, Brazil) or a semi-synthetic hypercholesterolemic diet (0.5% w/w cholesterol), based on a Western-type diet made of 20% fat, 0.5% cholic acid, 16.5% casein, vitamins and minerals according to American Institute of Nutrition AIN-93 recommendations (20). Both dietary groups were divided into 3 subgroups (*n* = 6 per group) and received an intravenous injection of PBS (vehicle group), 5 × 10^5^ particles/Kg of body weight of Phe-MCMN-Zn, or 5 × 10^5^ particles/Kg of body weight of scFv-anti-LDL(-)-MCMN-Zn nanoformulation (corresponding to 5 mg of scFv/Kg of body weight). The leukocyte-endothelium interaction (rolling and adhered cells) was observed in microcirculation of the cremaster muscle at baseline, 10, 30, and 60 min after the injections. Vascular permeability was analyzed 1 h after a further injection of FITC-albumin (50 mg/kg, 100 μL, Sigma-Aldrich, EUA) with an optical microscope (Axioplan II, Carl-Zeiss, Germany) and a video camera (ZVS, 3C75DE, Carl-Zeiss, Germany). The images were analyzed with the AxioVision 4.8 software. Blood collected one after finishing the intravital microscopy protocol from the cava vein into microtubes containing 10% EDTA for total leukocyte count and differential analysis with May Grumwald-Giemsa (Sigma-Aldrich, USA) staining. Plasma was separated by centrifugation and cholesterol and triglyceride levels were measured with commercial reagent kits (N°. 76 and N°. 87, respectively, Labtest, Brazil). Urine samples were collected by direct bladder puncture and analyzed with reagent strips (Urofita 10DL, Prodimol, Brazil) for the presence of erythrocytes, leukocytes, urobilinogen, bilirubin, protein, nitrite, ketones, pH and density.

### Effect of Nanoformulation on Atherosclerosis Progression

At 12 weeks of age, *Ldlr*^−/−^ male mice were allocated (*n* = 10/group) to receive weekly injections of PBS (vehicle group), scFv (5 mg of scFv/Kg of body weight), 5 × 10^5^ particles/Kg of body weight of Phe-MCMN-Zn, or 5 × 10^5^ particles/Kg of body weight of scFv-anti-LDL(-)-MCMN-Zn nanoformulation (corresponding to 5 mg of scFv/Kg of body weight). A control group (CCD-negative control) was fed chow diet without further treatment. Mice were fed a standard chow for a week before receiving a semi-synthetic hypercholesterolemic diet (0.5% w/w cholesterol) for 8 weeks. Water and diet were provided *ad libitum*. The first dose of each treatment was administered 1 week before starting the hypercholesterolemic diet. After 8 weeks of treatment, the atherosclerotic lesions at the aortic arch of mice were evaluated by ^18^F-FDG PET/CT imaging (Figure 6A). After imaging acquisition, mice were anesthetized with 10 mg/Kg of ketamine hydrochloride (1.0 g/10 mL) and 5 mg/Kg of xylazine hydrochloride (2.0 mg/100 mL) and exsanguination was done by cardiac puncture. Total blood leukocyte number was determined with Turk solution and the differential cell counting was done with Romanowsky staining. The urine was collected by bladder puncture for further analysis. Hearts were collected and prepared for morphometric analysis (*n* = 5/group) as previously described (21) and thoracic aortas were removed and stored in 4% formalin to determine their lipid content according to (22). The fragmented aortas were stained with Oil Red O, and the protein content of the tissue samples was evaluated by the BCA method and used for normalization of the data. The sections were analyzed with a Nikon optical microscope coupled to a camera; the image was captured using the program NIS-Elements AR (tm) version 3.10 (Nikon, USA). The Image J image analyzer software (version for Mac, NIH, USA) was used for lesion area measurements. Results are expressed as μmol of lipid/μg of protein.

### PET/CT Molecular Imaging

Computed associated tomography (CAT) and Positron emission tomography were (PET) were performed according to Mateo et al. (23) and conducted in 12-h-fasted mice. After blood glucose was measured by glucometer (Contour TS, Bayer, Mishwaka, USA), mice were briefly anesthetized with isoflurane inhalation and FDG (19.35 ± 4.19 MBq) was administered intravenously by ocular plexus. Isoflurane was removed and animals came back to conscience status. After 3 h, mice anesthetized again with isoflurane inhalation and subjected to small-animal PET imaging (Albira microPET-SPECT-CT, Bruker Biospin, Billerica, USA), wich was acquired during 30 min. Computed associated tomography was acquired with 400 projections, 400 μA and 45 KeV. CAT images were reconstructed with FBP algorithm (Filtered BackProjection), resulting in 125 mm isometric voxels. Maximum Likelihood Estimation Method (MLEM) algorithm was used on the reconstruction process of PET images. Every reconstruction steps was done with Albira Reconstructor software (5.6 VERSION). Fused images of PET and CAT were obtained with PMOD Fusion software (3.3.07 VERSION) (PMOD Technologies, Zurich, Switzerland). After coregistration, volumes of interest (VOI) were built around aortic arc using CAT images. The same VOI was applied to PET images to calculate standardized uptake value (SUV) values (23).

### Statistical Analysis

Data were analyzed by ANOVA and the results are reported as mean values ± standard deviations (SD). Tukey's test was used for paired comparisons analysis. A value of *p* < 0.05 was considered statistically significant.

## Results

### Synthesis Optimization of scFv-anti-LDL(-)-MCMN-Zn Nanoformulation

#### Pre-Formulation Study to Obtain Lecithin-Lipid-Core Nanocapsules

The lecithin-lipid-core nanocapsules showed a white-turbid macroscopic aspect. All formulations presented pH values slightly acid (Supplementary Table 2). Laser diffraction analysis showed unimodal size distribution profiles when lecithin was used up to 9 mg/mL (Supplementary Figure 1). The volume-weighted mean diameters D[3,4]v were similar (*p* > 0.05) (close to 125 nm) when lecithin was used from 3 to 9 mg/mL, while lecithin above 10 mg/mL promoted D[3,4]v higher than 1,400 nm (Supplementary Figure 2). The size distributions showed narrow polydispersity for LNC_3_ to LNC_9_, but broad polydispersity for LNC_10_ to LNC_14_ (Supplementary Table 2). Furthermore, the median diameters by number of particles, d(0.5)_n_, were lower than 90 nm for all formulations, and zeta potential decreased from −8.2 mV (LNC_3_) to −20.0 mV (LNC_9_) remaining constant for the other formulations. LNC_9_ was the selected lecithin-lipid-core nanocapsule formulation. Dynamic light scattering analysis of this formulation (Supplementary Figure 3) showed a mean hydrodynamic diameter (z-average) of 117±4 nm and a polydispersity index (PDI) of 0.1.

#### Pre-Formulation Study to Obtain Chitosan-Coated Lecithin-Lipid-Core Nanocapsules

Chitosan (5 to 14 mg/mL) reacted with lecithin-lipid-core nanocapsules (LNC_9_) (Supplementary Figure 4). The pH values varied from 3.6 to 4.0, D[3,4]v values were close to 130 nm, except for that prepared with the lowest concentration of chitosan, which D[3,4]v was 161 nm (Supplementary Table 3). Laser diffraction analysis showed unimodal particle size distributions (Supplementary Figure 5), with a polydispersity of 1, excepting for ${\text{LNC}}_{0.5}^{+}$, which value was 2. Zeta potential values were positive from 9.6 to 17.6 mV, using chitosan solution from 5 to 8 mg/mL, and from 16.0 to 19.2 mV using chitosan solution from 9 to 12 mg/mL. It was higher than 20 mV using chitosan solution above 13 mg/mL. New batches of ${\text{LNC}}_{0.5}^{+}$, ${\text{LNC}}_{0.7,}^{+}$ and ${\text{LNC}}_{1.4}^{+}$ were prepared and analyzed by laser diffraction (Supplementary Figure 6). After storage at 5°C, a higher kinetic stability was observed for ${\text{LNC}}_{0.7}^{+}$ (Supplementary Table 4). ${\text{LNC}}_{0.7}^{+}$ prepared using a 7 mg/mL chitosan solution in 1% acetic acid showed pH of 4.1 ± 0.0, D[3,4]v of 130 ± 3 nm and a polydispersity of 1 (Supplementary Figure 7). Zeta potential was 15.0 ± 1.8 mV, and the hydrodynamic mean diameter (z-average) was 127 ± 3 nm with PDI of 0.2. All physico-chemical parameters were similar (*p* > 0.05) to the former batches of ${\text{LNC}}_{0.7}^{+}$, excepting pH (*p* < 0.05), which was higher for the latter since a lower concentration of acetic acid was used to disperse chitosan. ${\text{LNC}}_{0.7}^{+}$ (prepared using 1% acetic acid) was analyzed by nanoparticle tracking analysis (NTA) (Supplementary Figure 8) showing hydrodynamic diameter, median diameter and particle number density, respectively, of 162 ± 11 nm, 161 ± 8 nm, and (0.94 ± 0.4) ×10^13^ particles/mL.

#### Pre-Formulation Study to Obtain scFv-anti-LDL(-) Functionalized Nanocapsules

The surface functionalization of chitosan-coated lecithin-lipid-core nanocapsules with scFv-anti-LDL(-) was carried out by forming an organometallic complex with Zn^+2^ (Supplementary Figure 9). To determine the minimal concentration of scFv to passivate the surface of the nanocapsules, a pre-formulation study was performed. Four different formulations of multi-wall Zn^+2^(MCMN-Zn)-nanocapsules reacted with scFv-anti-LDL(-) at 50, 100, 200, and 300 μg/mL, using Zn^+2^ at 25 μg/mL. Dynamic light scattering analysis (Supplementary Figure 10) showed similar correlation decays for the formulations containing scFv-anti-LDL(-) higher than 100 μg/mL but a slower decay for that containing it at 50 μg/mL. Despite a moderate polydispersity (PDI = 0.2) determined for all batches, the mean diameters (z-average) (Supplementary Figure 11) were 347 ± 122, 123 ± 5, 124 ± 9, and 121 ± 8 nm for the scFv-anti-LDL(-)-MCMN-Zn nanoformulations containing the scFv at 50, 100, 200, and 300 μg/mL, respectively. The quantification of scFv in the ultrafiltrate (non-bound to the nanocapsule surface) using a calibration curve (5 to 70 μg/mL) (Supplementary Figure 12) showed that the non-bound amount of scFv (soluble in the continuous phase of the nanoformulation) was lower than 15% for the nanoformulations containing 200 and 300 μg/mL (Table 1).

**Table 1:** Quantification of non-bound scFv-anti-LDL(-) in the ultrafiltrate of scFv-anti-LDL(-)-MCMN-Zn nanoformulation containing different concentrations of scFv.

**scFv concentration in the nanoformulation**	**Non-bound scFv-anti-LDL(-)**	
	**μg/mL**	**%**
50 μg/mL	32.6 ± 4.4	65.3 ± 8.8
100 μg/mL	19.1 ± 9.1	19.0 ± 9.1
200 μg/mL	21.7 ± 14.3	10.8 ± 7.1
300 μg/mL	41.8 ± 6.1	13.9 ± 2.0

### Evidence of Intracellular Uptake of scFv-anti-LDL(-)-MCMN-Zn Nanoformulation *in vitro*

**Figure 1:**
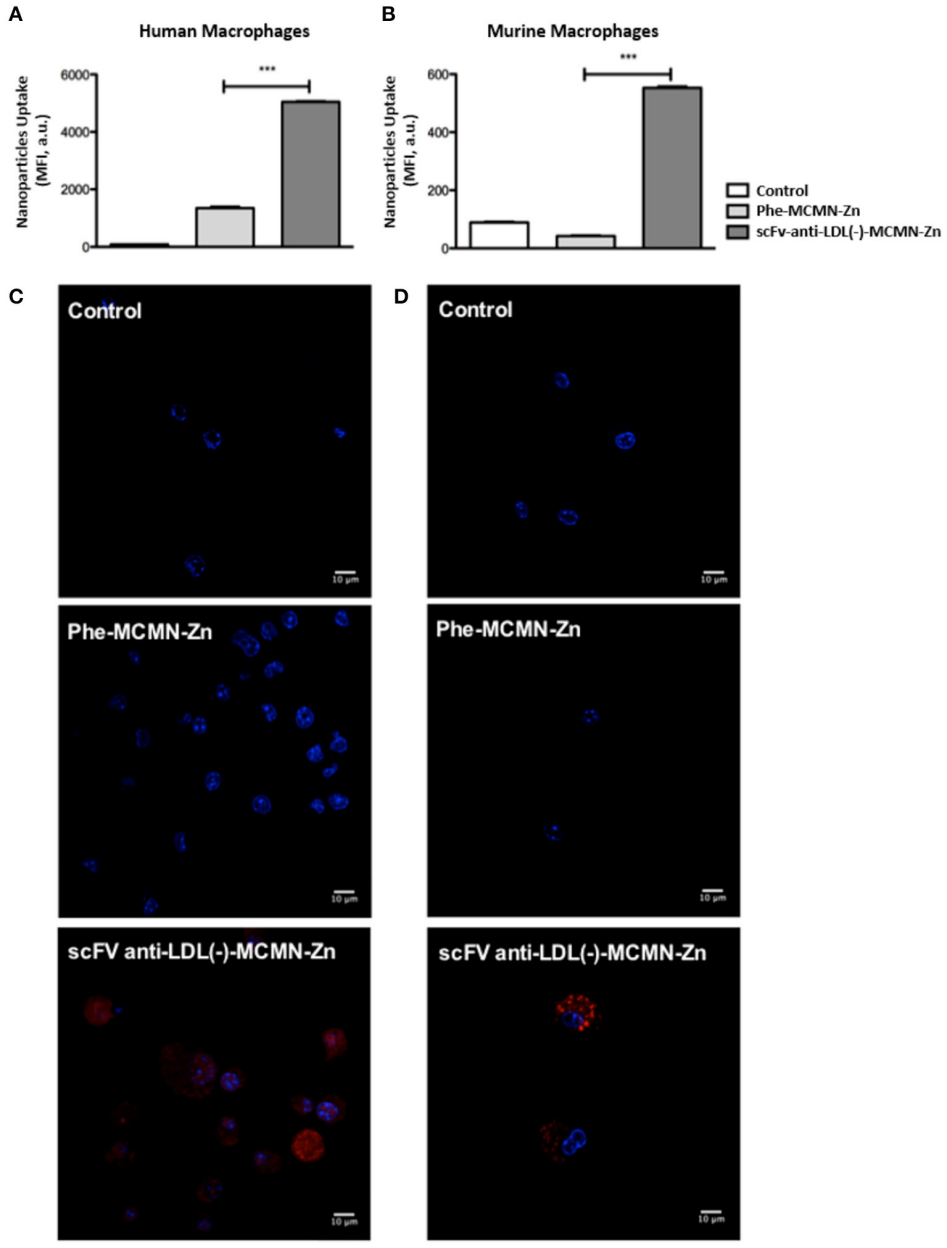
Internalization of the scFv-anti-LDL(-)-MCMN-Zn nanoformulation by human and murine primary macrophages. **(A,B)** Flow cytometry measurements data from human **(A)** and murine **(B)** primary macrophages treated with 10^4^ particles/mL of scFv-anti-LDL(-)-MCMN-Zn nanoformulation (containing 6.25 μg/mL of scFv) for 3 h. **(C,D)** Representative confocal microscopy images of human **(C)** and murine **(D)** macrophages exposed to the same conditions. Nanocapsules were labeled with Rhodamine B (red fluorescence) and macrophages nuclei stained with DAPI (blue fluorescence) (63× magnification, scale bar = 10 μm). Data from three independent experiments, performed in triplicate, are expressed as the means ± SD; ***p < 0.001 compared with Control; ANOVA followed by the Tukey-Kramer's test.

**Figure 2:**
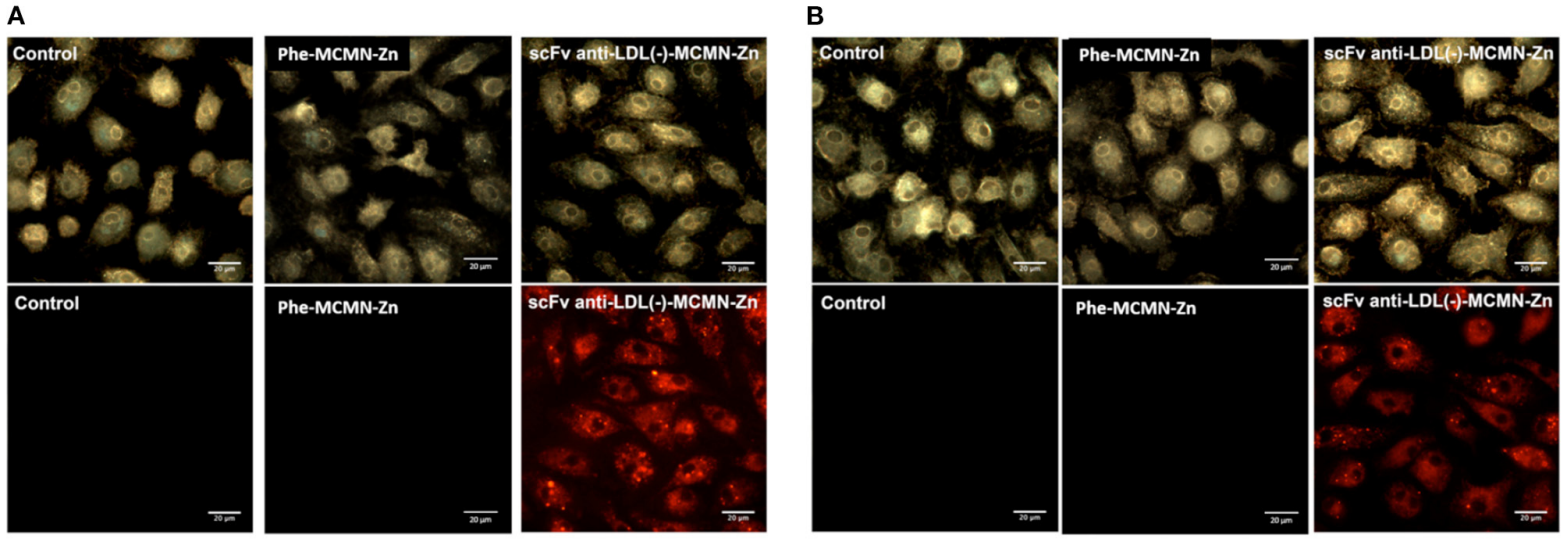
Internalization of the scFv-anti-LDL(-)-MCMN-Zn nanoformulation, by human and murine primary macrophages, monitored by enhanced dark-field microscopy (upper images; CytoViva®) and fluorescence (lower images; red emission). Data from **(A)** human and **(B)** murine macrophages treated 3 h with 10^4^ particles/mL of non-targeting Phe-MCMN-Zn or scFv-anti-LDL(-)-MCMN-Zn nanoformulation (containing 6.25 μg/mL of scFv) conjugated with Rhodamine B. Control: non-treated macrophages (100× magnification images).

Flow cytometry analysis showed that both human (Figure 1A) and murine (Figure 1B) primary macrophages significantly internalized scFv-anti-LDL(-)-MCMN-Zn nanoformulation, after 3 h of incubation (*p* < 0.001). The intracellular uptake of scFv-anti-LDL(-)-MCMN-Zn nanoformulation *in vitro* was confirmed by confocal analysis (Figures 1C,D) and by fluorescence, enhanced dark-field and hyperspectral CytoViva® microscopy (Figure 2; Supplementary Video 1—control of human macrophages, Supplementary Video 2—control of murine macrophages, Supplementary Video 3—human macrophages treated with scFv-anti-LDL(-)-MCMN-Zn, and Supplementary Video 4—murine macrophages treated with scFv-anti-LDL(-)-MCMN-Zn). The fluorescence microscopy images monitoring the typical red emission of Rhodamine B clearly showed the presence of nanocapsules inside macrophages, whereas the enhanced dark-field CytoViva® microscopy images showed the presence of a large number of bright yellowish spots in the cell cytoplasm corresponding to the nanocapsules, as compared to the control, whose spectrum (Supplementary Figure 13) registered using the CytoViva® microscope hyperspectral mode exhibiting a maximum at 600 nm also corroborated those results.

### Identification of Endocytosis Mechanisms Related to the Internalization of scFv-anti-LDL(-)-MCMN-Zn Nanoformulation by Macrophages

**Figure 3:**
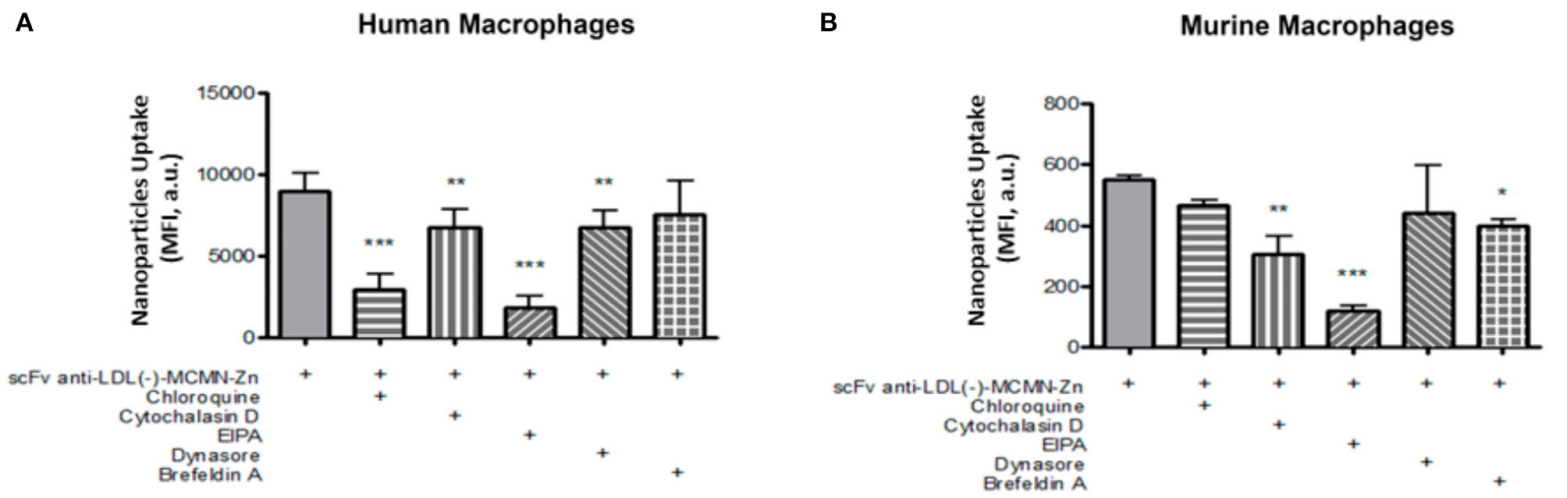
Endocytosis mechanisms involved in the internalization of the scFv-anti-LDL(-)-MCMN-Zn nanoformulation by human and murine primary macrophages. Flow cytometry measurements data from **(A)** human and **(B)** murine macrophages treated with 10^4^ particles/mL of scFv-anti-LDL(-)-MCMN-Zn nanoformulation (containing 6.25 μg/mL of scFv) of scFv-anti-LDL(-)-MCMN-Zn nanoformulation for 3 h, after 1 h incubation with 50 μM Chloroquine, 40 μM Cytochalasin D, 50 μM EIPA [5-(N-Ethyl-N-isopropyl) amiloride], 80 μM Dynasore, or 1 μM Brefeldin A. Data from three independent experiments, performed in triplicate, are expressed as the means ± SD; ***p < 0.001, **p < 0.01 and *p < 0.05 compared with scFv-anti-LDL(-)-MCMN-Zn nanoformulation without any inhibitor; ANOVA followed by the Tukey-Kramer's test.

Macropinocytosis inhibitor EIPA profoundly inhibited nanoformulation internalization both in human (79.4% inhibition, *p* < 0.001) and murine (79.5% inhibition, *p* < 0.001) macrophages (Figures 3A,B). The uptake was also significantly inhibited by actin polymerization inhibitor Cytochalasin D in both cell types (25.2% and 44.7% inhibition for human and murine macrophages respectively, *p* < 0.01). Reduced nanoformulation uptake by human macrophages was also detected in response to the inhibitor of lysosomal acidification Chloroquine (67.4% inhibition, *p* < 0.001) and the inhibitor of dynamin, Dynasore (25.5% inhibition, *p* < 0.01), while ER to Golgi traffic inhibitor Brefeldin A attenuated nanocapsule uptake in murine (28.1% inhibition, *p* < 0.05), but not human macrophages.

### Effect of scFv-anti-LDL(-)-MCMN-Zn Nanoformulation on LDL(-) Uptake by Macrophages

**Figure 4:**
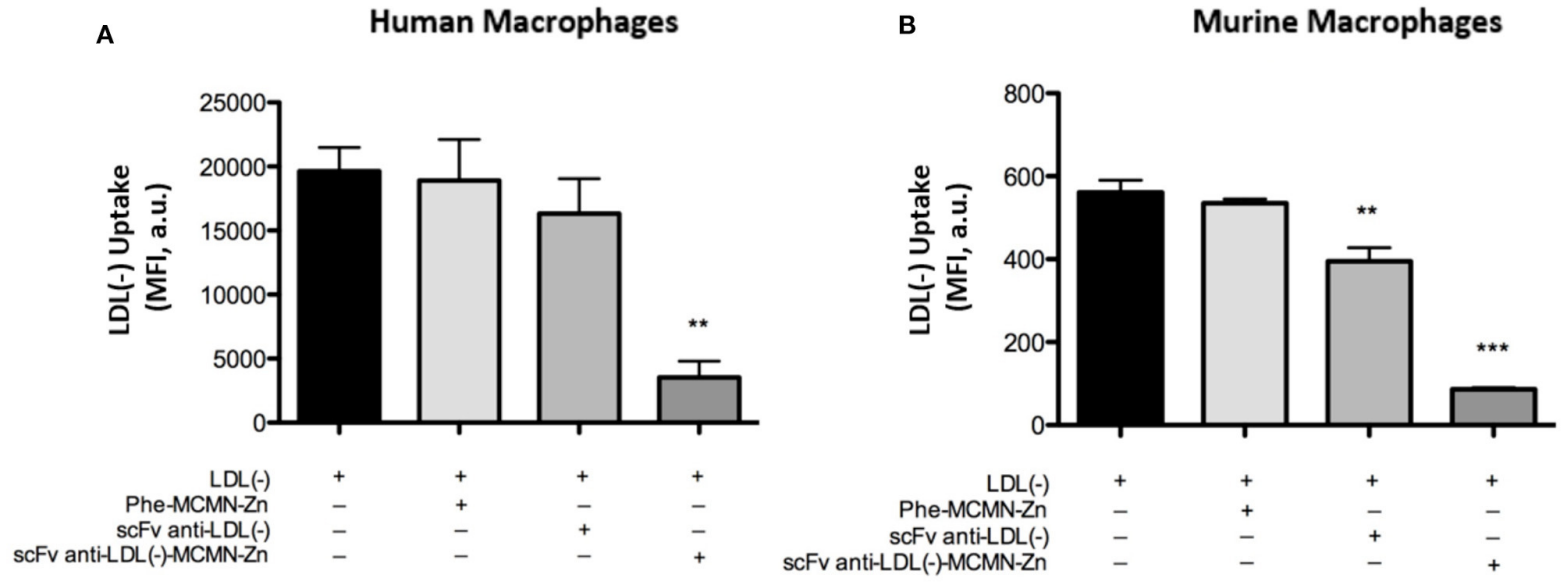
Effects of the scFv-anti-LDL(-)-MCMN-Zn nanoformulation on LDL(-) uptake by human and murine primary macrophages. Flow cytometry measurements data from **(A)** human and **(B)** murine macrophages treated with 10^4^ particles/mL of scFv-anti-LDL(-)-MCMN-Zn nanoformulation (containing 6.25 μg/mL of scFv) of scFv-anti-LDL(-)-MCMN-Zn nanoformulation and 37.5 μg/mL of LDL(-) individually or combined for 3 h. LDL(-) was fluorophore DiI(C_18_)-labeled. Data from three independent experiments, performed in triplicate, are expressed as the means ± SD; **p < 0.01 ***p < 0.001 compared with LDL(-); ANOVA followed by the Tukey-Kramer‘s test.

The treatment with 10^4^ particles/mL of scFv-anti-LDL(-)-MCMN-Zn (containing 6.25 μg of scFv/mL of nanoformulation) for 3 h significantly decreased LDL(-) uptake by both human (*p* < 0.01) and murine (*p* < 0.001) macrophages (Figures 4A,B, respectively) compared to LDL(-) control.

### Effect of scFv-anti-LDL(-)-MCMN-Zn Nanoformulation on the Pro-inflammatory Effects of LDL(-) in Macrophages

**Figure 5:**
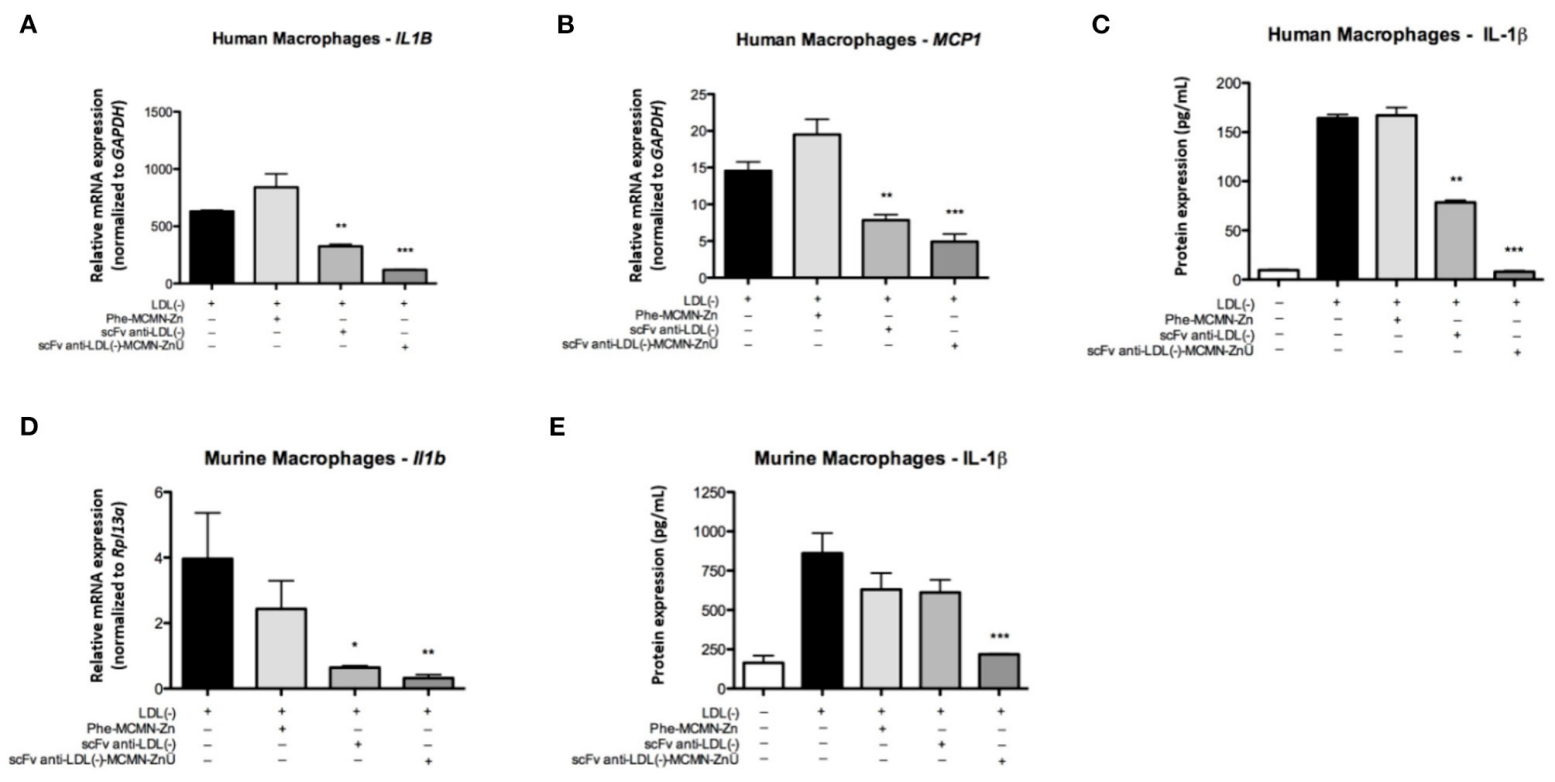
Effects of the scFv-anti-LDL(-)-MCMN-Zn nanoformulation on pro-inflammatory cytokines in human and murine primary macrophages stimulated by LDL(-). **(A,B)** mRNA levels of *IL1B* **(A)** and *MCP1/CCL2* **(B)** in human macrophages. **(C)** Protein levels of IL-1β on human macrophages. **(D,E)** Transcriptional **(D)** and protein **(E)** expression of *Il1b* in murine macrophages. Data expressed as the mean ± SD, **p < 0.01 and ***p < 0.001 compared with LDL(-). ANOVA followed by the Tukey-Kramer's test. *P < 0.05.

Gene expression analysis of human macrophages showed that the treatment with 10^4^ particles of scFv-anti-LDL(-)-MCMN-Zn (containing 6.25 μg of scFv/mL of nanoformulation) plus LDL(-) reduced the mRNA levels of *IL1B* (Figure 5A) and *MCP1/CCL2* (Figure 5B) when compared to LDL(-) control. The treatment also decreased IL-1β protein levels (Figure 5C) after 48 h of stimulation. In murine macrophages, the nanoformulation significantly diminished *Il1b* mRNA expression and abolished IL-1β protein secretion (Figures 5D,E) compared to LDL(-).

### Safety Evaluation of scFv-Anti-LDL(-)-MCMN-Zn Nanoformulation

The evaluation of the endothelium-leukocyte interaction determined by leukocytes rolling to vascular endothelium (24) indicated that either 5 × 10^5^ particles/Kg Phe-MCMN-Zn nanocapsules or 5 × 10^5^ particles scFv-anti-LDL(-)-MCMN-Zn nanoformulation (corresponding to 5 mg of scFv/Kg of body weight) did not affect the number of rolling leukocytes in normocholesterolemic (Supplementary Figure 14A) as well as in hypercholesterolemic mice (Supplementary Figure 14B) when compared to the PBS-treated controls (*p* > 0.05). The same pattern was observed for leukocyte adhesion (Supplementary Figures 15A,B). Moreover, the intravenous injection of both Phe-MCMN-Zn and scFv-anti-LDL(-)-MCMN-Zn nanoformulation did not induce hemolysis, thrombus formation or hemorrhagic areas in the microcirculation. Furthermore, as indicated by lack of FITC-albumin extravasation from blood vessels, the scFv-anti-LDL(-)-MCMN-Zn nanoformulation did not increase vascular permeability in normo and hypercholesterolemic *Ldlr*^−/−^ mice as compared to the control group (Supplementary Figure 16). The blood cells count of the studied groups indicated that the absolute number of leukocytes, neutrophils, monocytes and eosinophils were at the reference range for wild-type (25) and the *Ldlr*^−/−^ mice (26) without differences among the studied groups (Supplementary Table 5). The urianalysis did not show the presence of blood or erythrocytes in the urine indicating the absence of intravascular hemorrhage in all studied groups (Supplementary Table 6). Moreover, no traces of protein were found in the urine suggesting that neither Phe-MCMN-Zn nanocapsules nor scFv-anti-LDL(-)-MCMN-Zn nanoformulation cause glomerular damage in mice. These findings reinforce our previous data showing no signs of acute toxicity or end-organ lesion induced by this nanoformulation (21). Overall, these findings demonstrate the safety of the scFv-anti-LDL(-)-MCMN-Zn nanoformulation intravenously administered at 5 mg/kg dose used to treat *Ldlr*^−/−^ mice in this study.

### Effects of the scFv-Anti-LDL(-)-MCMN-Zn Nanoformulation on the Development of Atherosclerotic Lesions of *Ldlr*^–/–^ Mice

**Figure 6:**
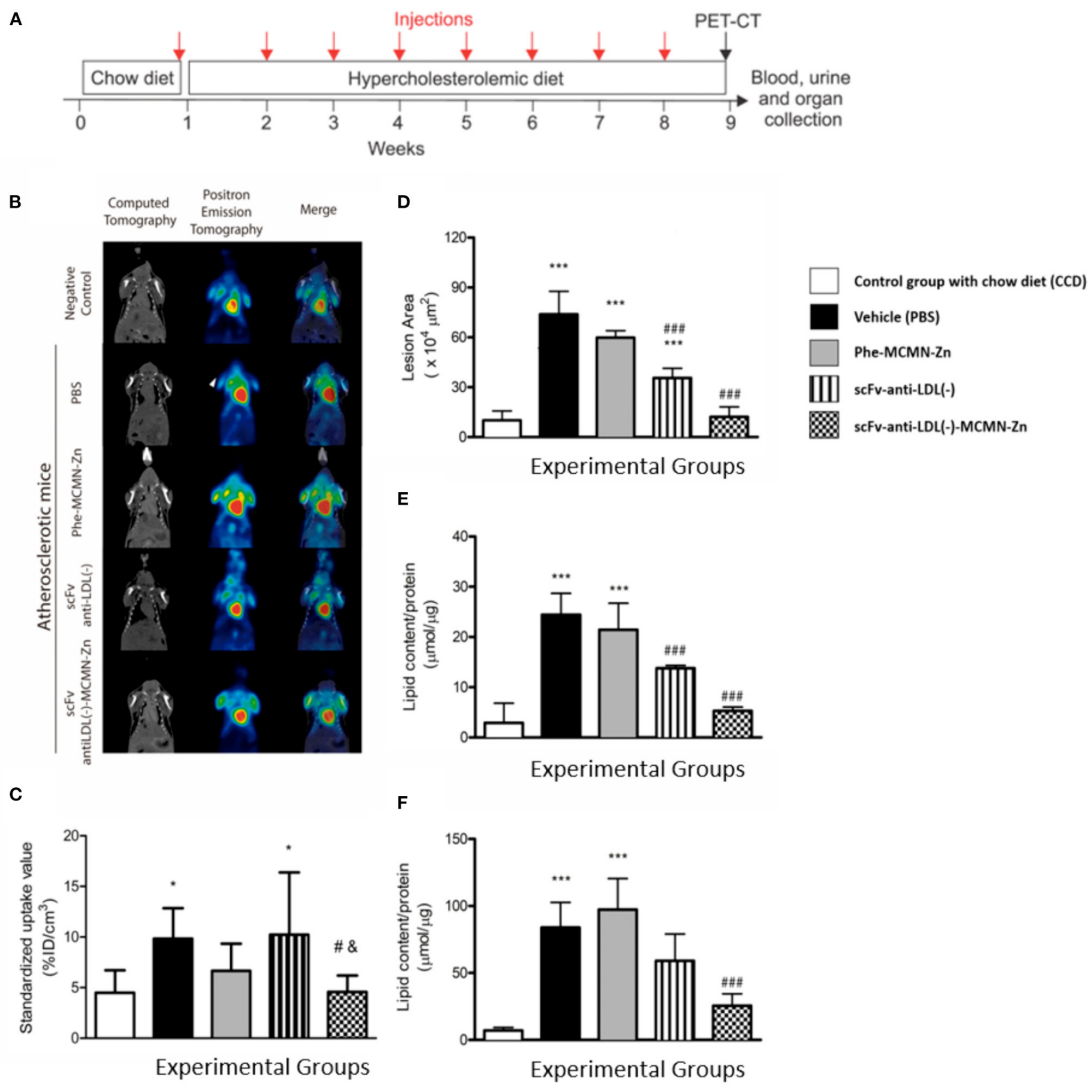
Weekly intravenous administration of scFv-anti-LDL(-)-MCMN-Zn nanoformulation prevents atherosclerosis development. **(A)** Scheme showing the experimental protocol; 12-week-old male *Ldlr*^−/−^ mice were fed a semi-synthetic hypercholesterolemic diet (0.5% w/w cholesterol). Mice were divided into 5 groups (*n* = 5 per group) and received weekly injections of PBS (vehicle group), 5 × 10^5^ particles/Kg of body weight of Phe-MCMN-Zn nanocapsules, 5 mg scFv-anti-LDL(-)/Kg of body weight and scFv-anti-LDL(-)-MCMN-Zn nanoformulation [corresponding to 5 mg of scFv-anti-LDL(-)/Kg of body weight]. A control group was fed chow diet without any treatment (CCD). After 8 weeks of treatment, the atherosclerotic lesions at the aortic arch of mice were evaluated by ^18^F-FDG PET/CT imaging. Blood, urine and heart were collected to further analysis. **(B,C)** Representative coronal ^18^F-FDG PET/CT images **(B)** and standardized uptake value analysis **(C)** of the ^18^F-FDG retention in the aortic arc region. **(D)** Quantification of the atherosclerotic lesion areas (× 10^4^ μm^2^) in the aortic arch of *Ldlr*^−/−^ mice. **(E,F)** Measurement of lipid content in the aorta **(E)** and in the aortic arch **(F)** of *Ldlr*^−/−^ mice. *p < 0.05 and ***p < 0.001 in relation to CCD control group; ^#^*p* < 0.01 and ^###^*p* < 0.001 compared to the PBS group and ^&^*p* < 0.01 compared to scFv-anti-LDL(-)-treated group.

PET-CT molecular imaging was used to evaluate ^18^F-FDG retention in the aortic arc region as the accumulation of inflammatory cells into atherosclerotic lesions can increase intraplaque glucose metabolism reflecting in higher FDG signal (23). As expected, ^18^F-FDG absorption in aortic arc was greater in PBS-treated mice fed a hypercholesterolemic diet when compared to standard chow-fed counterparts (negative control) (9.85 ± 0.99; 4.49 ± 0.83, respectively). No change in ^18^F-FDG retention into the aortic arc region was observed with mice treated with Phe-MCMN-Zn nanocapsules, and scFv-anti-LDL(-) when compared to PBS group. In contrast, scFv-anti-LDL(-)-MCMN-Zn treatment significantly decreased ^18^F-FDG retention into aortic arc region compared to PBS (4.57 ± 0.57; 9.85 ± 0.99, respectively) or scFv-anti-LDL(-) treated animals (4.57 ± 0.57; 10.22 ± 2.17, respectively), indicating a lower local inflammation (Figures 6B,C). Accordingly, the morphometric analysis of atherosclerotic lesions at the aortic arch (Figure 6D) as well as the lipid content of the aortas and aortic arch (Figures 6E,F) of *Ldlr*^−/−^ mice also showed that the scFv-anti-LDL(-)-MCMN-Zn nanoformulation was more effective to inhibit the atherosclerosis progression than the scFv-anti-LDL(-) non-derivatized on the surface of the nanoparticles.

## Discussion

One of the greatest advantages of the use of nanotechnology in medical applications is the ability to engineer materials that can interact with different cell features and specific substrates in order to deliver drugs or bioactive molecules. Moreover, the use of nanodevices conjugated with antibody fragments, such as the scFv (27), has been increased due to their small size, compared to whole antibodies, which enables the reduction of the diameter variation of the nanoparticulate systems after conjugation (28).

Recently, we proposed a new strategy to functionalize the surface of polymeric nanocapsules using an organometallic complex to bind a ligand to the nanostructure with high yields without needing further purification steps (21, 29). While the proportions of triglyceride, sorbitan monostearate and PCL have been previously studied for the polysorbate 80-coated lipid-core nanocapsules (30), the present study described the optimized proportions of lecithin, chitosan, metal ion and ligand [scFv-anti-LDL(-)] to produce the scFv-anti-LDL(-)-MCMN-Zn nanoformulation. Considering the volume-weighted mean diameters D[3,4]v, the polydispersity (SPAN) and zeta potential values for lecithin-lipid-core nanocapsules, its optimal concentration is 9 mg/mL. The results are in accordance with those previously observed (31) for lecithin adsorbed on poly(lactide)-nanostructures, which zeta potential values reached a plateau after surface site saturation. The negative zeta potential is a consequence of the oxygenated species at the particle-water interface, which value increased in modulus with the increase in the lecithin concentration due to the presence of phosphatidic acid as an impurity. The selected formulation (LNC_9_) was then reacted with chitosan at different concentrations. The highest volume-weighted mean diameter and the highest polydispersity (SPAN) determined for the formulation prepared with the lowest concentration of chitosan (5 mg/mL) suggested that the surface coating of the nanocapsules was not complete. Furthermore, a plateau is observed when the 7 mg/mL chitosan solution was used to coat the LNC_9_ formulation suggesting that a surface site saturation is occurring, and a monolayer is achieved. After storage at 5°C, only the formulation coated with chitosan solution at 7 mg/mL was kinetically stable. Considering the presence of polysorbate 80 in the formulation, the mechanism of stability of those colloidal particles is based on both steric hindrance (by the non-ionic surfactant) and electrostatic repulsion (by the polycationic polymer) at the particle-water interface. To decrease the concentration of acetic acid in the formulation, a new batch of ${\text{LNC}}_{0.7}^{+}$ was obtained by using a 7 mg/mL chitosan solution in 1% acetic acid. All physicochemical characteristics were similar between the ${\text{LNC}}_{0.7}^{+}$ formulations, except for the pH, which was higher for that prepared with lower amounts of acetic acid. Previously, scFv-anti-LDL(-)-MCMN-Zn nanoformulations have been proposed using 3 mg/mL of lecithin, 0.5 mg/mL of chitosan and 200 μg/mL of scFv (20). Here, we proposed to optimize the composition to increase the kinetic stability of the nanocapsules. Indeed, the chitosan-lecithin-coated lipid-core nanocapsules prepared with 9 mg/mL lecithin and 0.7 mg/mL chitosan showed constant mean diameter for 21 days (stored at 5°C). Therefore, after varying the concentration of lecithin and chitosan at the surface of the nanocapsules, a new pre-formulation study to determine the optimal concentration of scFv to passivate the interface particle-water was necessary. The lack of reproducibility in producing the scFv-anti-LDL(-)-MCMN-Zn nanoformulation at 50 μg/mL of scFv showed a non-compliance of the product, which was discarded. Moreover, the quantification of non-bound scFv to the nanocapsule surface indicated that its optimal concentration in the nanoformulation is higher than 200 μg/mL as nanoformulations prepared at 200 and 300 μg/mL showed percentages of scFV bound to the nanocapsule surface higher than 85%. For this reason, the scFv-anti-LDL(-)-MCMN-Zn nanoformulation at 300 μg/mL was selected for the biological evaluations.

Here, we have found that only the nanocapsules functionalized with the scFv-anti-LDL(-) were efficiently internalized by murine and human macrophages compared to the control formulation. Consistent with our findings, previous studies (32, 33) have shown that conjugated nanoparticles present greater cellular uptake, sustained intracellular retention and, therefore, better efficacy in cells when compared to unconjugated nanoparticles. To deliver drugs or molecules that might be unstable in physiological conditions and maintain its intracellular levels, the nanoparticles should be able to cross key barriers, such as the cell membranes (34), which typically prevent the entrance of large molecules despite the occurrence of endocytosis (35). Nanoparticles interact differently with the cells compared to molecules in general (36) and the mechanisms involved in their internalization by cells, are deeply influenced by their physicochemical properties as well as by their surface ligands (37).

Generally, phagocytosis occurs in specialized cells like monocytes, macrophages, neutrophils and dendritic cells in order to protect the organism from infectious agents or to uptake solid particles, such as drug delivery nanoparticles (38). The key steps in this process are the opsonization involvig opsonins proteins (immunoglobulins and complement proteins) (39) and the formation of the phagosome, which is able to transport the particle throughout the cytoplasm to fuse with lysosomes and form a phagolysosome. The phagolysosomes become acidified and acquire an enzymatic content that is essential for polymer biodegradability of synthetic nanoparticles and can allow drug release (40). While particles need a minimum size of 500 nm to undergo phagocytosis (41), size is not the only determinant characteristic in this process and surface properties must be also considered (42).

Unlike phagocytosis, endocytosis mechanisms are present in almost all cell types and include: macropinocytosis, clathrin- and caveolin-mediated endocytosis, both dependent on dynamin recruitment, as well as other clathrin- and caveolin-independent mechanisms (37). Macropinocytosis occurs in many cells, including macrophages, and involves the formation of protrusions that merge with each other and fuse with the cell membrane (43). Although macropinocytosis does not have any selectivity (44) and engulfs large particles, with no specific surface coating, generating macropinosomes bigger than 1 μm (45), it is considered one of the best routes for drug delivery (46). Other endocytic processes as clathrin- and caveolin-mediated endocytosis (47, 48) can also be involved in the uptake of the nanoparticles by cells.

The data presented here support that the internalization of the scFv-anti-LDL(-)-MCMN-Zn nanocapsules occurs via phagocytosis (demonstrated by the Cytochalasin D-mediated inhibition of the polymerization of actin filaments) and mainly by macropinocytosis (about 80% of inhibition of the nanoformulation internalization by EIPA). Nanoparticle internalization pathways may be related to several factors. For instance, nanoparticles prepared with the same material and originated from the same solution or suspension may differ in size, shape and porosity, interacting differently with cells and inducing particular endocytic mechanisms (49). The adsorption of proteins or peptides on the surface of nanoparticles may also alter their unique properties, such as size and charge (50). While different cell types present distinguished internalization strategies (51), the pharmacological inhibitors commonly used do not generally present high specificity for each mechanism and may influence alternative internalization routes (52). In our study, the shape and surface are constant for the nanocapsule formulation. Thus, the size distribution is likely to be the most relevant parameter influencing the uptake mechanism. Considering that the majority of the scFv-anti-LDL(-)-MCMN-Zn nanoformulation is taken up by macrophages via macropynocytosis, one of the best routes for drug delivery (46), this nanodevice may be used for new therapeutic approaches, combining the functionalization of nanoparticles and encapsulation of specific drugs at one system to target drug delivery to the site of disease.

Cardiovascular diseases are the leading worldwide cause of death (53), which explains why nanotechnology has been increasingly applied to the understanding, diagnosis and treatment of these pathologies (54). In the context of atherosclerosis, it is important to reduce cholesterol accumulation into the arterial wall to prevent foam cell formation (8). Of note, our findings clearly show that the scFv-anti-LDL(-)-MCMN-Zn nanoformulation promoted a significant decrease of LDL(-) uptake by both human and murine macrophages. The internalization of the complex LDL(-)-scFv-anti-LDL(-)-MCMN-Zn nanoformulation, that occurs predominantly by phagocytosis and macropinocytosis, somehow could transiently disturb the uptake of LDL(-) by the scavenger and/or other cell receptors. This is in agreement with previous reports demonstrating that the *in vitro* treatment of macrophages with a complex of poly(latic acid)-nanoparticles conjugated to an anti-apoB-100 monoclonal antibody significantly decreased the LDL uptake by these cells (55).

An important observation derived from our *in vivo* findings is the vascular safety of the scFv-anti-LDL(-)-MCMN-Zn nanoformulation administered intravenously. In fact, this nanoformulation did not induce hemolysis, or changes in leukocytes-endothelium interactions and in vascular permeability in mice fed standard or hypercholesterolemic diets. Moreover, as previously reported (21), no signs of acute toxicity or end-organ lesions were observed in *Ldlr*^−/−^ mice treated with this nanoformulation. Besides being safe, weekly intravenous administration of scFv-anti-LDL(-)-MCMN-Zn nanoformulation in *Ldlr*^−/−^ mice led to smaller atherosclerotic lesions at the aortic arch as well as reduced lipid content of total aorta, supporting the anti-atherosclerotic action of our nanoformulation. Furthermore, the inflammatory process in the atherosclerotic plaque was also inhibited by the treatment of *Ldlr*^−/−^ mice with the nanoformulation as shown by F^18^-FDG PET/CT imaging. It is worth to highlight that the derivatization of the anti-LDL(-) on the surface of the Zn^+2^(MCMN-Zn)-nanocapsules significantly enhanced the atheroprotective action of the anti-LDL(-) antibody fragment.

These *in vivo* atheroprotective actions may be explained by the decrease of the uptake of atherogenic lipoproteins by macrophages that is a crucial step for atheroma formation and atherosclerosis progression. Moreover, the inflammatory response inside the atherosclerotic plaque is in great part promoted by macrophages being determinant for lesion progression, instability and rupture. In fact, the LDL(-) pro-inflammatory action may play a key role in atherosclerosis due to its effect on endothelial cells, stimulating the production of several cytokines, chemokines and growth factors related to the atherosclerotic process (56). In macrophages, the LDL(-) promotes the secretion of biologically active IL-1β through two main mechanisms: the activation of CD14 and TLR4 receptors, known as priming, and the activation of NRLP3 inflammasome, with the formation of the inflammasome complex NLRP3-ASC and the activation of caspase-1 (57, 58). IL-1β acts as an important mediator of the inflammatory response in cell proliferation and differentiation processes (59) and has direct functions in the formation and stability of the atherosclerotic plaque by inducing the production of proteolytic enzymes by macrophages, endothelial cells and smooth muscle cells, presenting great importance on spreading inflammation to other cell types (60). It has been shown that IL-1β receptor-deficient mice have smaller atherosclerotic lesions (~33%) compared to mice without the deletion of this gene, reinforcing its importance in promoting atherosclerosis (61). In turn, MCP-1, presents an important role in the regulation of migration and infiltration of monocytes from the circulation through the vascular endothelium during the inflammatory response not only at the beginning of the subendothelial migration but also in the amplification of this process (62). Recent studies involving human monocytes from atherosclerotic plaque reported increased levels of MCP-1 compared to those isolated from peripheral blood monocytes (63). Increased MCP-1 expression was also observed in the aortic arch of hypercholesterolemic rabbits (64), associated with the increase of TNF-α (tumor necrosis factor-α), VCAM-1 (vascular cell adhesion molecule 1) and IL-1β (65). Therefore, MCP-1 has also been considered as a potential marker of the atherogenic process (66, 67). Thus, the inhibition of MCP-1 and MCP-1 levels induced by the scFv-anti-LDL(-)-MCMN-Zn shows its inhibitory action against the proinflammatory effects of LDL(-) in the atherosclerotic plaques.

In summary, this study showed that an optimized scFv-anti-LDL(-)-MCMN-Zn nanoformulation is taken up by macrophages in a major extent via phagocytosis and macropinocytosis, can suppress LDL(-)-mediated accumulation and production of pro-inflammatory factors involved in the atherosclerotic process. Altogether, our findings point out the potential of the scFv-anti-LDL(-)-MCMN-Zn nanoformulation as a promising strategy for early intervention in atherosclerosis to prevent the development and progression of this disease, mainly in drug-resistant hyperlipidemic patients with high levels of LDL(-). In addition, the results pave the way to further investigations of the nanoformulation as a nanocarrier for drug delivery and targeting.

## Data Availability Statement

The original contributions presented in the study are included in the article/Supplementary Material, further inquiries can be directed to the corresponding author/s.

## Ethics Statement

The study was approved by the Animal Research Ethics Committee, for the use of C57BL/6J homozygous low-density lipoprotein receptor-deficient mice (Ldlr-/-) (n. 392/2013) of the Faculty of Pharmaceutical Sciences from the University of São Paulo, in agreement with the Brazilian College for Animal Experimentation guidelines.

## Author Contributions

MC, AP, and DA: manuscript draft, study conception and design. SF, SG, BB, DN, AP, and DA: manuscript revision. MC, WT, MU, KA, SF, DN, AP, and DA: data analyses. MC, MA, WT, MK, MU, AAs, AAl, CD, MS, SK, and MB: material preparation and data collection. All authors contributed to the article and approved the submitted version.

## Conflict of Interest

The authors declare that the research was conducted in the absence of any commercial or financial relationships that could be construed as a potential conflict of interest.
