# Supplementary material for: scFv-Anti-LDL(-)-Metal-Complex Multi-Wall Functionalized-Nanocapsules as a Promising Tool for the Prevention of Atherosclerosis Progression
Source: Front Med (Lausanne). 2021 Apr 20;8:652137. doi: 10.3389/fmed.2021.652137 (PMC8095373; doi:10.3389/fmed.2021.652137)
Supplement: Supplementary file 1 [file Data_Sheet_1.docx]

**Supplementary Material**

**Methods**

Synthesis optimization of lecithin-lipid-core nanocapsules

Seven formulations were prepared by interfacial deposition of polymer, varying the amounts of lecithin (0.03 g, 0.05 g, 0.08 g, 0.09 g, 0.10 g, 0.12 g e 0.14 g) in the formulation. An organic phase containing PCL (0.10 g), sorbitan monostearate (0.04 g), and capric-caprylic triglyceride (0.12 g) in acetone (25 mL) at 40 ºC was added of a lecithin-ethanol solution (10 mL) and after 5 minutes, the mixture was injected into an aqueous dispersion (50 mL) of polysorbate 80 (0.08 g). After the turbid solution was maintained under magnetic stirring for 10 minutes, the solvents were evaporated and the formulation was concentrated under reduced pressure at 40ºC to a final volume of 10 mL. Formulations were labeled considering their lecithin concentration: 3 mg/mL (LNC_3_), 5 mg/mL (LNC_5_), 8 mg/mL (LNC_8_), 9 mg/mL (LNC_9_), 10 mg/mL (LNC_10_), 12 mg/mL (LNC_12_) and 14 mg/mL (LNC_14_).

Chitosan coating optimization of lecithin-lipid-core nanocapsules

Different chitosan solutions in 2% acetic acid were prepared at 5 mg/mL, 7 mg/mL, 8 mg/mL, 9 mg/mL, 10 mg/mL, 11 mg/mL, 12 mg/mL, 13mg/mL and 14mg/mL and filtered, after dissolution, through a 0.45 μm membrane (MerckMillipore^®^, Darmstadt, DE). The resultant chitosan solution (1 mL) was added drop by drop to LNC_9_ (9 mL) under high magnetic stirring. The reaction was carried out for 2 hours. The selected formulation was prepared using 1% acetic acid. A 7 mg/mL chitosan solution in 1% acetic acid was added drop by drop to LNC_9_ (9 mL) under high magnetic stirring and maintained in reaction for 2 hours. This formulation (chitosan-lecithin-coated lipid-core nanocapsules) was used in the next steps of surface functionalization described below.

Production and characterization of scFv-anti-LDL(-):

The yeast was grown in BMMY (buffered methanol-complex) medium at 20°C for 72 hours at 200 rpm in an incubator shaker (#AG CH-4103, Minitron, Infors HT, Bottmingen, Switzerland), with the addition of 1% methanol and 1 mM PMSF every 24 hours. A Ni Sepharose 6 Fast Flow resin (#17-5318-01, GE Healthcare Life Sciences, Sao Paulo, Brazil) was used to purify the supernatant of this culture. For the elution of the 2C7 scFv-anti-LDL(-), a binding buffer containing 500 mM imidazole (Sigma-Aldrich, Sao Paulo, Brazil) was added to the system. An enzymatic deglycosylation was performed for the cleavage of high-mannose glycans if presented in the purified scFv-anti-LDL(-). The protocol suggested by the manufactures included the denaturation of the scFv-anti-LDL(-) with 0.5% sodium dodecyl sulfate (SDS) (Sigma-Aldrich, Sao Paulo, Brazil) and 0.04 M dithiothreitol (DTT) (Sigma-Aldrich, Sao Paulo, Brazil ) for 10 minutes at 100 °C, and a post 1-hour treatment with a 0.5% sodium citrate buffer containing 1000 units of endoglycosidase H (#P0702S, Endo H, New England Biolabs, MA, USA) at 37°C. Following electrophoresis of polyacrylamide gels, the characterization of the antibody fragment was performed as described in Kazuma et al, 2013, employing silver staining and Western blotting analysis (data not shown).

Surface functionalization of chitosan-lecithin-coated lipid-core nanocapsules using an organometallic complex to produce the scFv-anti-LDL(-)-nanoformulation

An aqueous solution of scFv-anti-LDL(-) at 462.39 µg/mL (A) was diluted with ultrapure water to produce solutions at 308.25 µg/mL (B), 154.13 µg/mL (C) and 77.07 µg/mL (D). Separately, zinc acetate (0.028 g) was dissolved in ultrapure water (10 mL), which was called solution E. Then, 9.75 mL of the selected formulation [chitosan-lecithin-coated lipid-core nanocapsules (LNC^+^_0.7_)] were added of solution E (0.25 mL) under magnetic stirring (900 rpm), which was called solution F. After 1 minute, 351.21 µL of solution F were poured into a vial to receive 648.79 µL of solution A under magnetic stirring (400 rpm). The procedure was repeated using the solutions B-D. In different vials, 351.21 µL of solution F were added one by one of 648.79 µL of solutions B, C and D under magnetic stirring (400 rpm). The reactions were carried out for 10 minutes producing 4 different formulations containing scFv-anti-LDL(-)-MCMN-Zn at 300, 200, 100 and 50 µg/mL, respectively, and Zn^+2^ at 25 µg/mL.

Physicochemical characterization of nanoparticles:

Particle size distribution curves were determined by laser diffraction (Mastersizer^®^ 2000 (Malvern Instruments, UK) after adding sufficient volume of the formulation in the wet unit containing about 150 mL of distilled water for an obscuration between 2% and 8%, and by dynamic light scattering (Zetasizer^®^ Nano-ZS, Malvern Instruments, UK) after diluting a sample of the formulation (500x) with ultrapure water (MilliQ^®^, MerckMillipore^®^, Darmstadt, DE). The polydispersity (SPAN) was calculated by dividing the difference of the values of diameters at the percentiles 90 and 10 by the value of the median diameter under the size distribution curve determined by laser diffraction. In addition, nanoparticle-tracking analysis (Nanosight LM10, NanoSight^®^, UK) was used to determine the hydrodynamic mean diameter and particle number density (concentration of nanocapsules determined by number of particles per milliliter of formulation). Zeta potential was determined by electrophoretic mobility in a ZetaPlus (Zeta Potential Analyser, Brookhaven Instruments Corporation, NY, USA) after diluting a sample of the formulation in 10 mmol/L NaCl aqueous solution. The pH values were measured at 25ºC directly in the formulation using a potentiometer (B474, Micronal, Sao Paulo, Brazil) previously calibrated at pH 4.0 and 7.0. The quantification of non-bound scFv-anti-LDL(-) in the nanocapsule formulation was carried out by the Lowry method for protein quantification after ultrafiltration-centrifugation (30 kDa Amicon^®^ Ultra, MerckMilipore^®^, USA) at 1,844 xg for 5 minutes. All measurements were conducted in triplicate batches of formulations (n=3).

Isolation of electronegative LDL:

Total LDL was separated from human blood plasma by sequential flotation ultracentrifugation at 56,000 xg for 7 hours at 4°C in a Optima XE-90 ultracentrifuge (Beckman Coulter, IN, USA). The LDL(-) was separated from the native LDL (nLDL) by fast protein liquid chromatography (FPLC, Bio-Rad Laboratories Inc, Hercules, CA, USA) using an ion exchange column (Sepharose UNO Q12, Bio-Rad Laboratories Inc, Hercules, CA, USA). The isolated LDL(-) fraction was dialyzed against phosphate buffered saline (PBS) and concentrated using a specific device (Vivaspin 20, 100,000 MWCO, GE Healthcare Life Sciences, Uppsala, Sweden).

Differentiation of human monocytes:

For differentiation into macrophages, monocytes were cultured for 6 days in serum-free medium (Macrophage-SFM, #12065074, Gibco^®^, Thermo Fisher Scientific, NY, USA) supplemented with 50 ng/mL human recombinant macrophage colony-stimulating factor (M-CSF, #78057, StemCell Technologies, WA, USA) at 37°C and 5% CO_2_ in fully humidified air.

Isolation of murine bone marrow cells and differentiation into macrophages:

Total bone marrow cells were isolated by flushing the bone marrow from both femur and tibia using RPMI 1640 medium (Gibco^®^, Thermo Fisher Scientific, MA, USA) supplemented with 10% fetal bovine serum (FBS) (Gibco^®^, Thermo Fisher Scientific, MA, USA). The isolated cells were passed through a cell strainer (70 µm size, BD Biosciences, CA, USA), centrifuged at 300 xg for 8 minutes at 4ºC, treated with a lysing buffer for lysing red blood cells, centrifuged again at the same conditions before counting. The isolated cells were differentiated for 6 days in RPMI 1640 medium supplemented with 30% of L929-conditioned medium, a source of murine M-CSF, and 10% fetal bovine serum (FBS, #16000044, Gibco^®^, NY, USA) at 37°C and 5% CO_2_ in fully humidified air.

Preparation of L929-conditioned medium:

Approximately 4.7 × 10^5^ L929 cells (NCTC clone 929, ATCC^®^ CCL-1™) were plated in a T-75-cm^2^ flask (Corning^®^, NY, USA) containing 55 mL of L929 medium (Eagle Minimum Essential Medium – EMEM – supplemented with 10% horse serum, ATCC^®^, WA, USA) and cultured at 37°C and 5% CO_2_ for 7 days. After this time, the supernatant was collected and filtered it through a 0.45 µm filter and 15 mL-aliquots were stored at -20°C.

Gene expression analysis by qRT-PCR:

Total RNA was isolated from 1 x 10^6^ cells using TRIzol reagent (Invitrogen^®^, Thermo Fisher Scientific, MA, USA), and 1 μg of total RNA was reverse-transcribed with the High Capacity RNA to cDNA Master Mix (Thermo Fisher Scientific, MA, USA), following manufacturer’s instructions. The real-time quantitative PCR reactions were performed with 20 ng of cDNA using the SYBR Green Master Mix (Thermo Fisher Scientific, MA, USA).

**Figures**


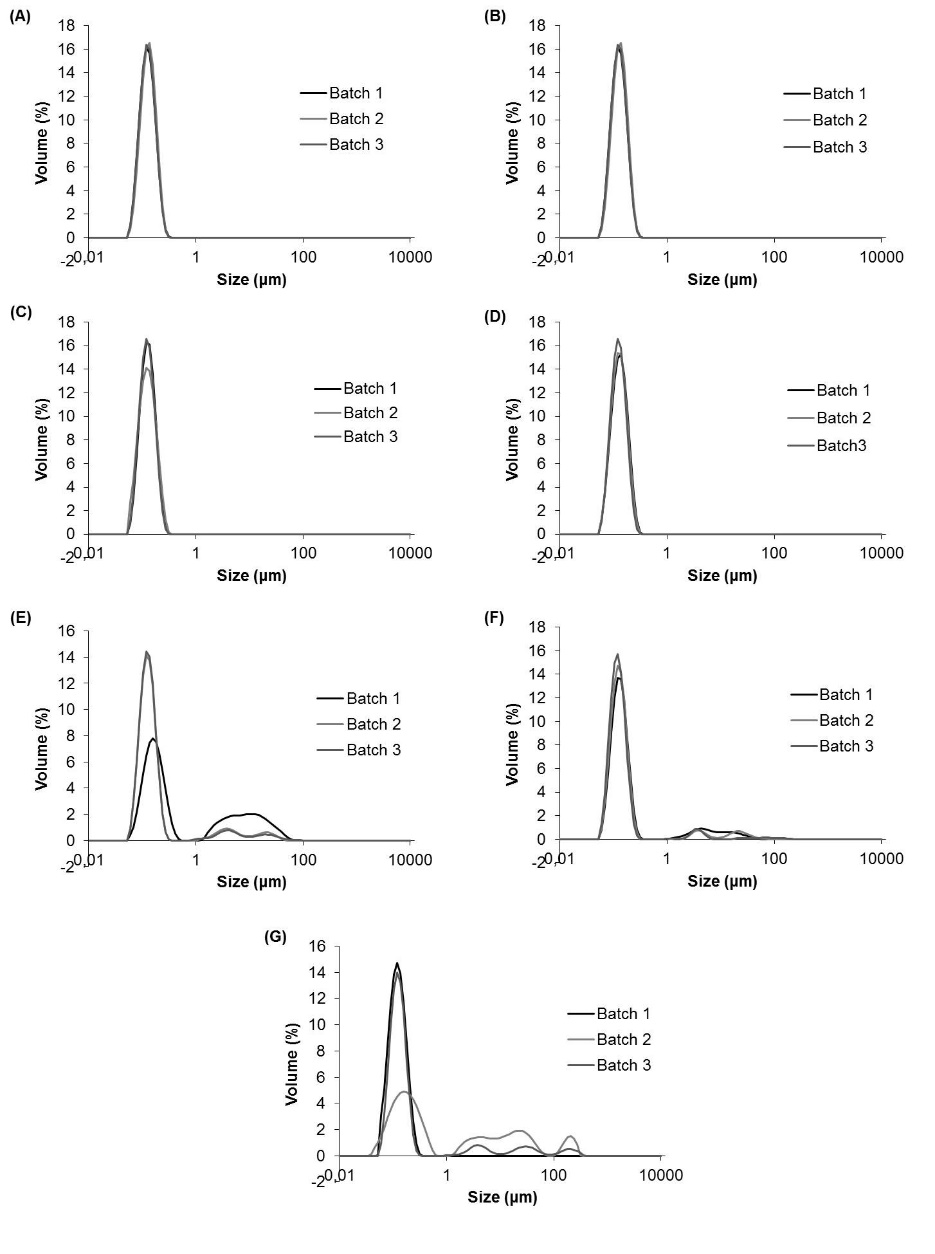


Figure S1. Particle size distribution by laser diffraction (Mastersizer 2000, Malvern, UK): (A) LNC_3_; (B) LNC_5_; (C) LNC_8_; (D) LNC_9_; (E) LNC_10_; (F) LNC_12_ and (G) LNC_14_, (n = 3).


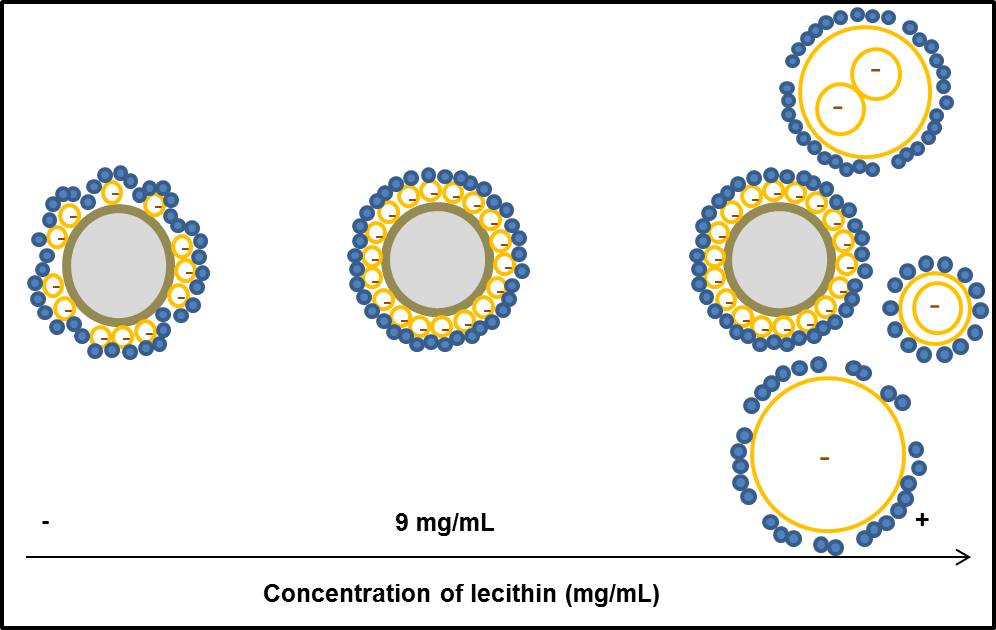


Figure S2. Illustrative model for the lecithin-lipid-core nanocapsules obtained using increased concentrations of lecithin. From the inner to the outer phase: the gray sphere represents the lipid-core, which is encapsulated by the polymeric wall (PCL) (green circle), stabilized by liposome-like structures (orange circles with a negative sign) and polysorbate 80-micelles (blue spheres). The best proportion was determined for lecithin at 9 mg/mL.


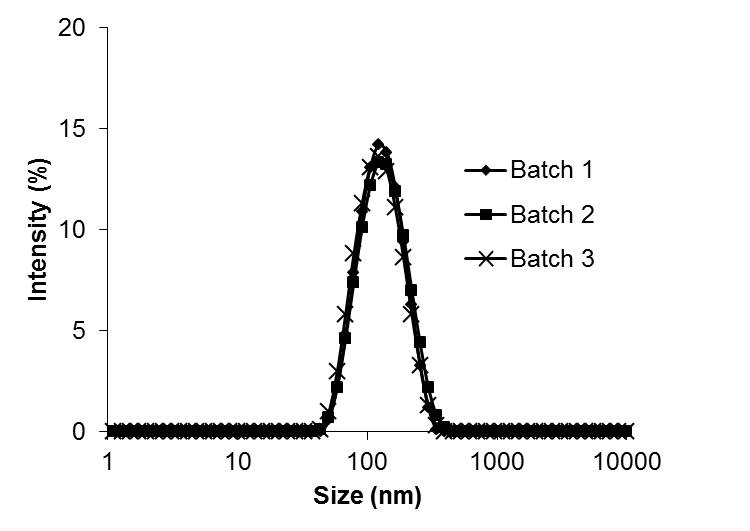


Figure S3. Particle diameter distribution by dynamic light scattering (Zetasizer ZS, Malvern, UK) for LNC_9_ (n = 3).


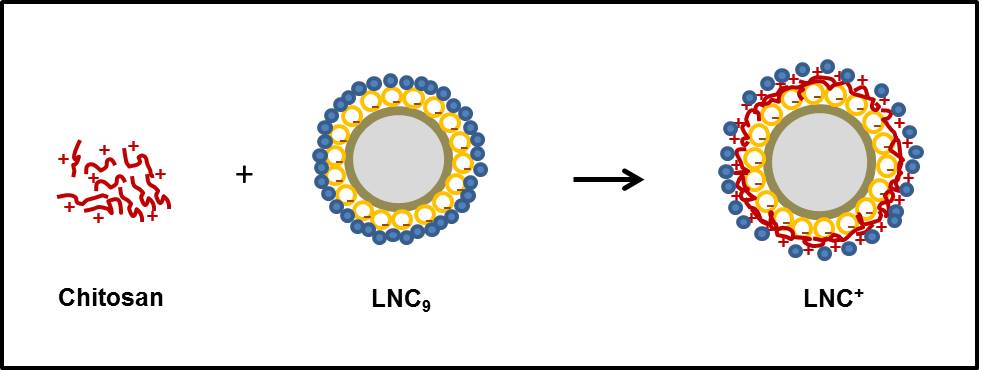


Figure S4. Illustrative model for the reaction of chitosan with lecithin at the surface of the lipid-core nanocapsules. Red lines represent the cationic polymer chitosan. LNC_9_, from the inner to the outer phase: the gray sphere represents the lipid-core, which is encapsulated by the polymeric wall (PCL) (green circle), stabilized by lecithin (orange circles with a negative sign) and polysorbate 80 micelles (blue spheres). LNC^+^, from the inner to the outer phase: the gray sphere represents the lipid-core, which is encapsulated by the polymeric wall (PCL) (green circle), coated by lecithin (orange circles with a negative sign) bound to chitosan (positive red lines). Polysorbate 80-micelles (blue spheres) is used as stabilizer.


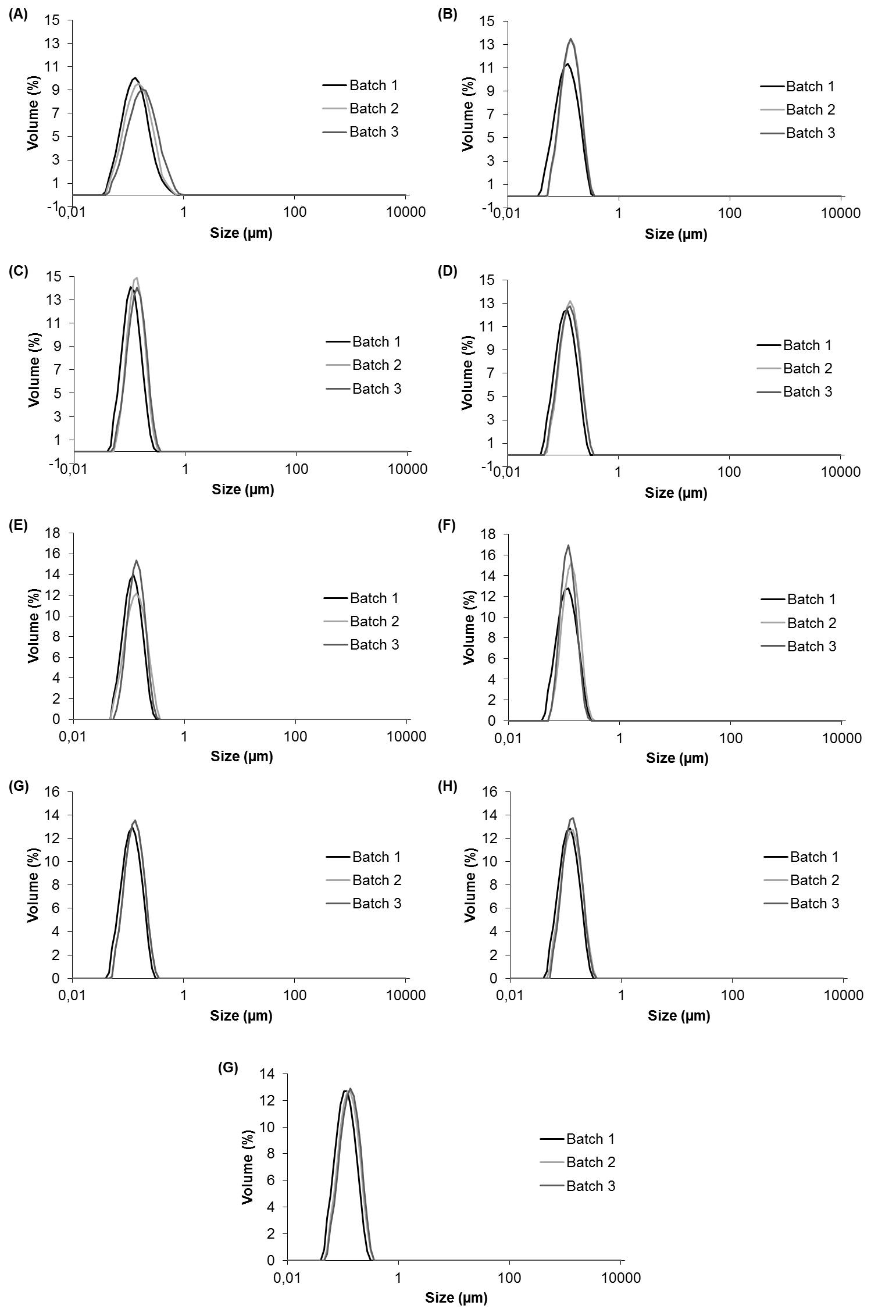


Figure S5. Particle size distribution by laser diffraction (Mastersizer 2000, Malvern, UK): (A) LNC^+^_0.5_; (B) LNC^+^_0.7_; (C) LNC^+^_0.8_; (D) LNC^+^_0.9_; (E) LNC^+^_1.0_; (F) LNC^+^_1.1_, (G) LNC^+^_1.2_, (H) LNC^+^_1.3_ and (I) LNC^+^_1.4_, (n = 3).


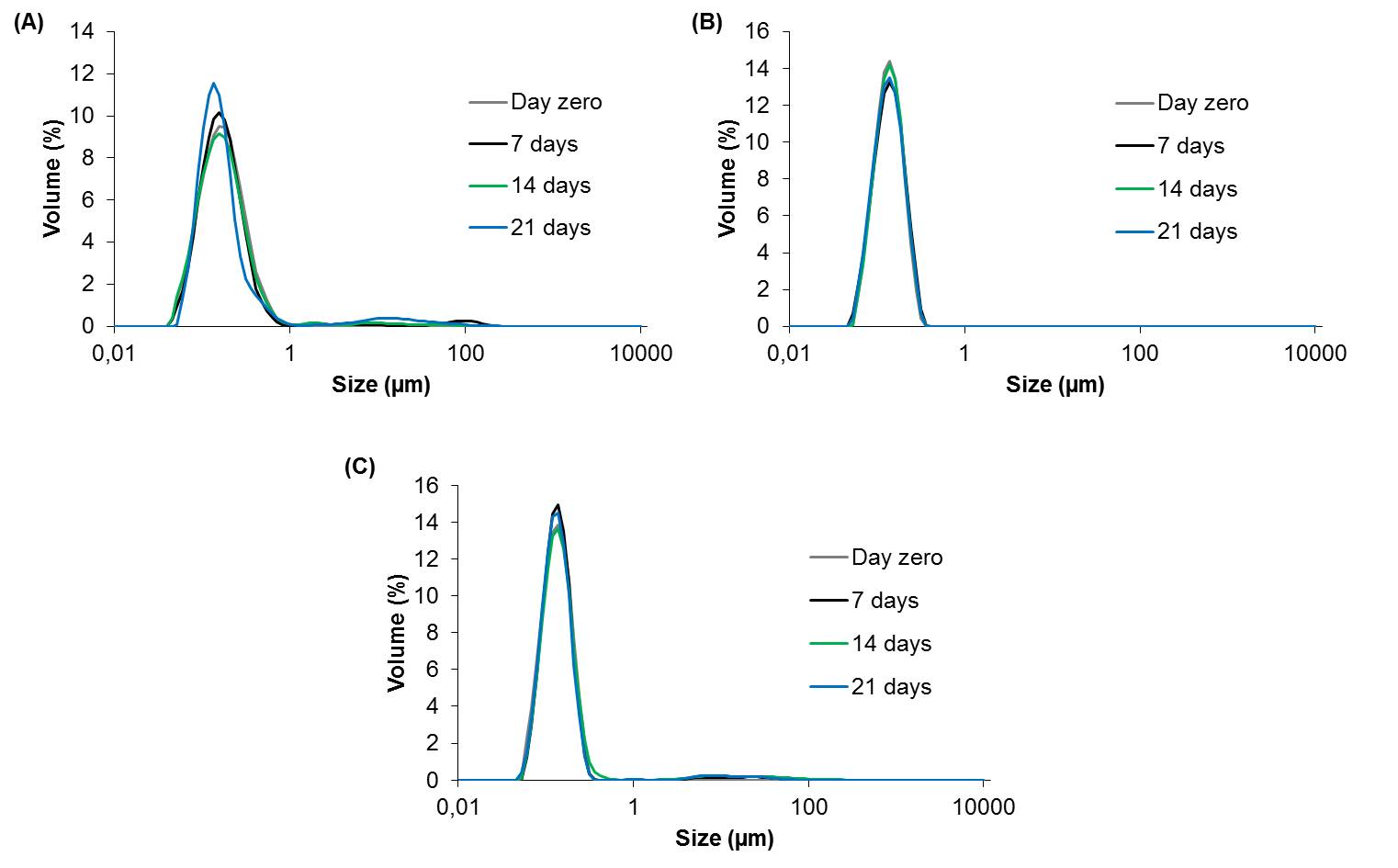


Figure S6. Particle size distribution by laser diffraction (Mastersizer 2000, Malvern, UK) immediately after preparation and after storage at 5±2°C for 7, 14 and 21 days: (A), LNC^+^_0.5_; (B), LNC^+^_0.7_ and (C), LNC^+^_1.4,_ (n=3).


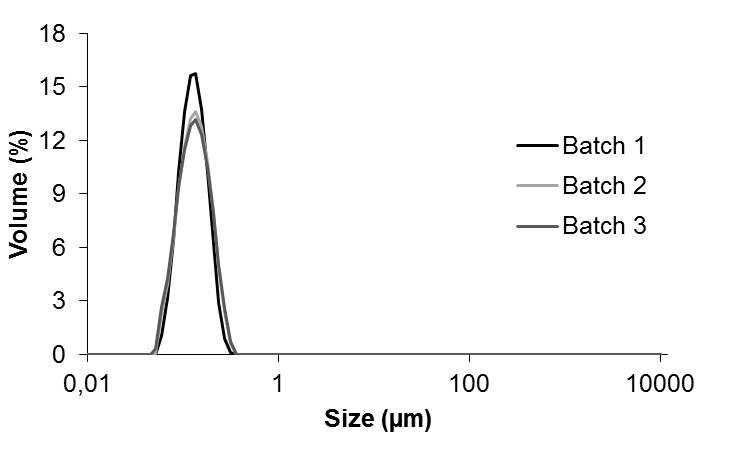


Figure S7. Particle size distribution by laser diffraction (Mastersizer 2000, Malvern, UK) of chitosan-lecithin-coated lipid-core nanocapsules (LNC^+^_0.7_) prepared using the chitosan solution at 7 mg/mL in 1% acetic acid.


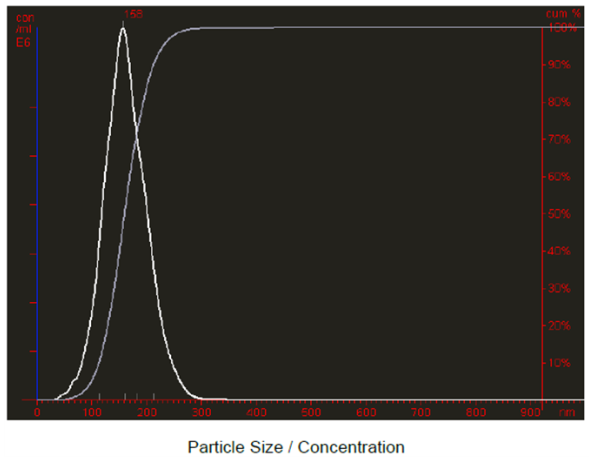


Figure S8. Particle size distribution by NTA (Nanosight^®^, Nanosight, UK), diameter as a function of the concentration of particles (% by number of particles), for chitosan-lecithin-coated lipid-core nanocapsules (LNC^+^_0.7_) prepared using the chitosan solution at 7 mg/mL in 1% acetic acid.


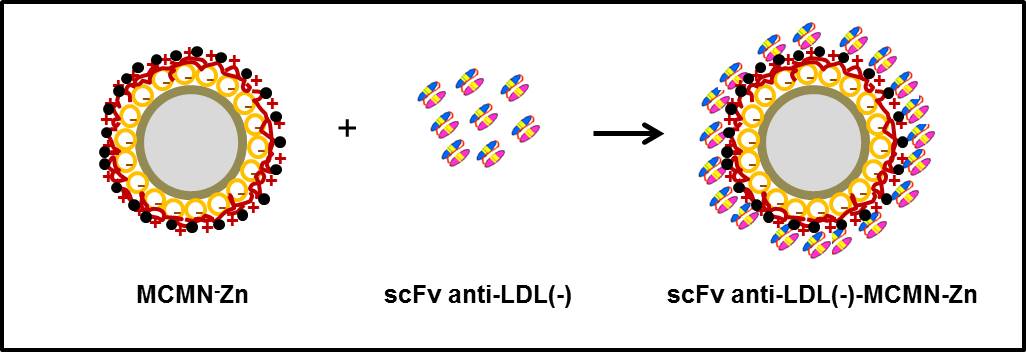


Figure S9. Illustrative model for the reaction of scFv-anti-LDL(-) and multi-wall nanocapsules to form the scFv-anti-LDL(-)-metal-complex multi-wall functionalized-nanocapsules. MCMN-Zn, from the inner to the outer phase: the gray sphere represents the lipid-core, which is encapsulated by the polymeric wall (PCL) (green circle), coated by lecithin (orange circles with a negative sign) bound to chitosan (positive red lines), which is complexed with zinc-II (black dots). scFv-anti-LDL(-) is represented by the blue and rose ellipses bonded together. scFv-anti-LDL(-)-MCMN-Zn, from the inner to the outer phase: the gray sphere represents the lipid-core, which is encapsulated by the polymeric wall (PCL) (green circle), coated by lecithin (orange circles with a negative sign) bound to chitosan (positive red lines), which is complexed with zinc-II (black dots), which is also complexed with scFv-anti-LDL(-) (blue and rose ellipses bonded together). The polysorbate 80-micelles were omitted to facilitate visualization of illustrative models.


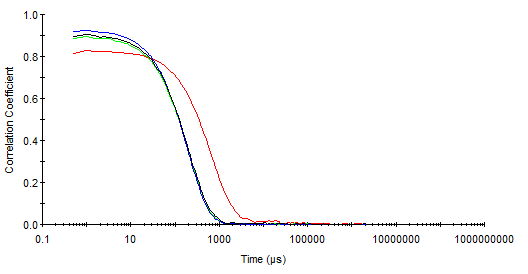


Figure S10. Correlation function by dynamic light scattering (Zetasizer ZS, Malvern, UK) for the nanocapsules complexed with different concentrations of scFv-anti-LDL(-): 50 µg/mL (red line), 100 µg/mL (green line), 200 µg/mL (black line), and 300 µg/mL (blue line).


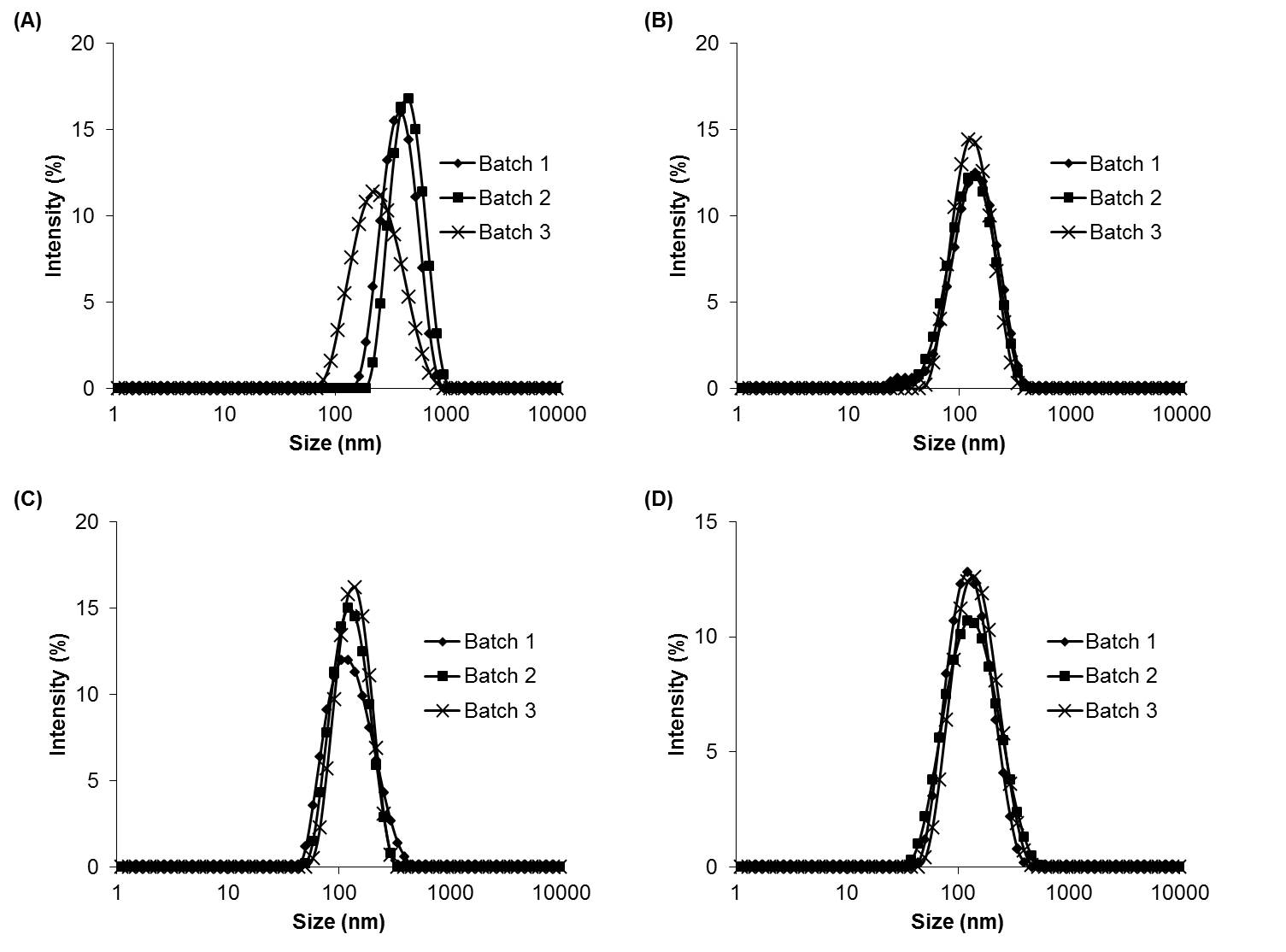


Figure S11. Particle diameter distributions by dynamic light scattering (Zetasizer ZS, Malvern, UK) for the multi-wall nanocapsules (MCMN-Zn) functionalized with scFv-anti-LDL(-) at 50, 100, 200 and 300 µg/mL, and Zn^+2^ at 25 µg/mL, respectively (A), (B), (C) and (D) prepared in triplicate batches (n= 3).


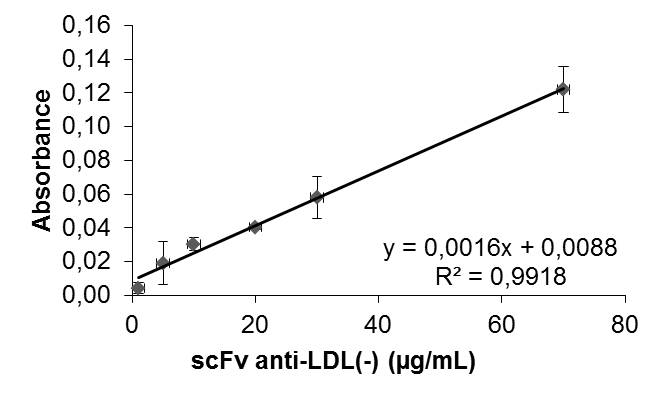


Figure S12. Calibration curve for scFv-anti-LDL(-) using the Lowry Method (n=3).


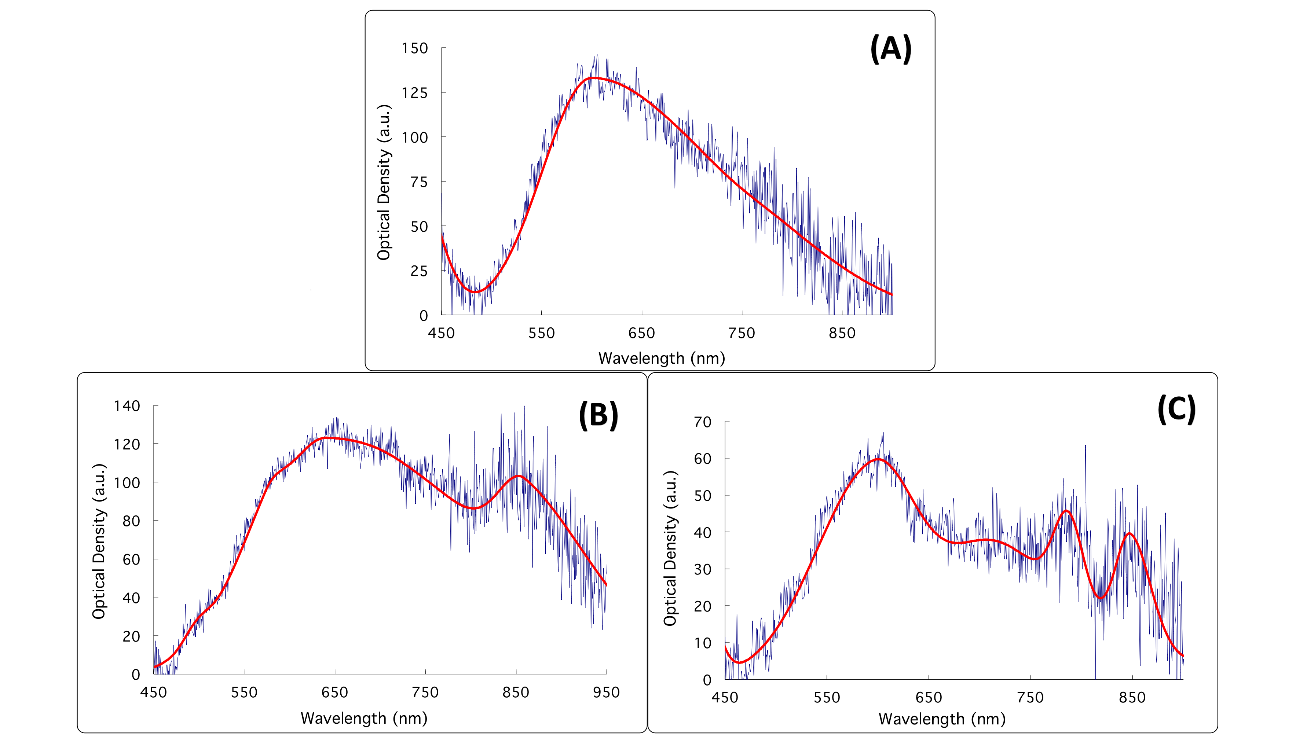


Figure S13. Mean spectrum of scFv-anti-LDL(-)-MCMN-Zn nanoformulation conjugated with Rhodamine B (A), and bright small areas inside of human (B) and murine (C) macrophages incubated with the same formulation in (A) for 3 hours. All spectra were acquired using the CytoViVa hyperspectral imaging system.


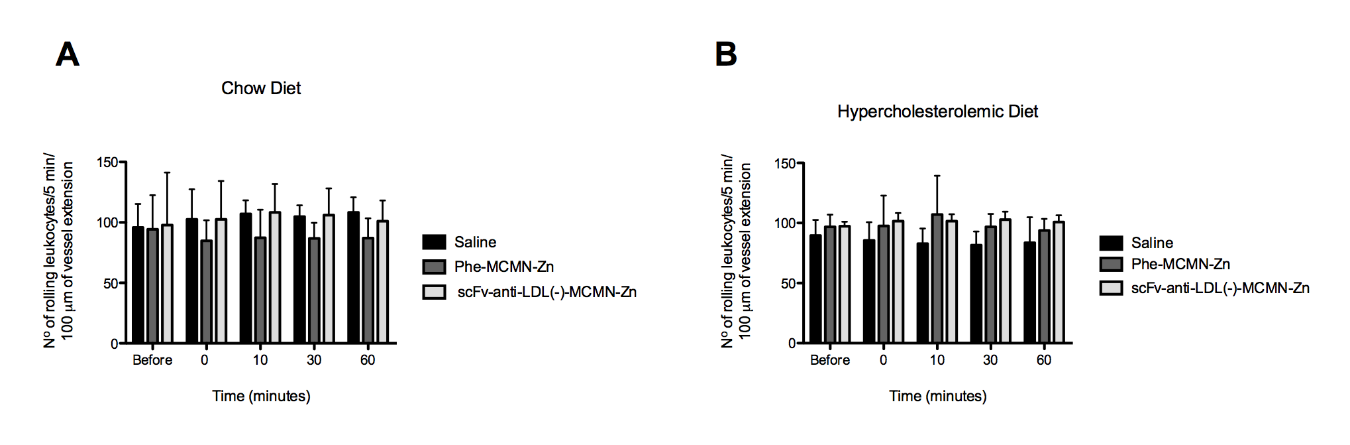


Figure S14. Effects of the intravenous injection of scFv-anti-LDL(-)-MCMN-Zn nanoformulation on the leukocyte-endothelium interactions. 12-week-old male *Ldlr^-/-^* mice previously fed with chow diet (A) or hypercholesterolemic diet (B) for 8 weeks received intravenous injection of 0.9% Saline, 5 x 10^5^ particles/Kg body weight of Phe-MCMN-Zn nanocapsules or 5 x 10^5^ particles/Kg body weight (corresponding to 5 mg scFv/Kg body weight) of scFv-anti-LDL(-)-MCMN-Zn nanoformulation. Number of rolling leukocytes in the microcirculation of the cremaster muscle was assessed by intravital microscopy at baseline, 10, 30 e 60 minutes after the injections. Data are expressed as mean ± SD.


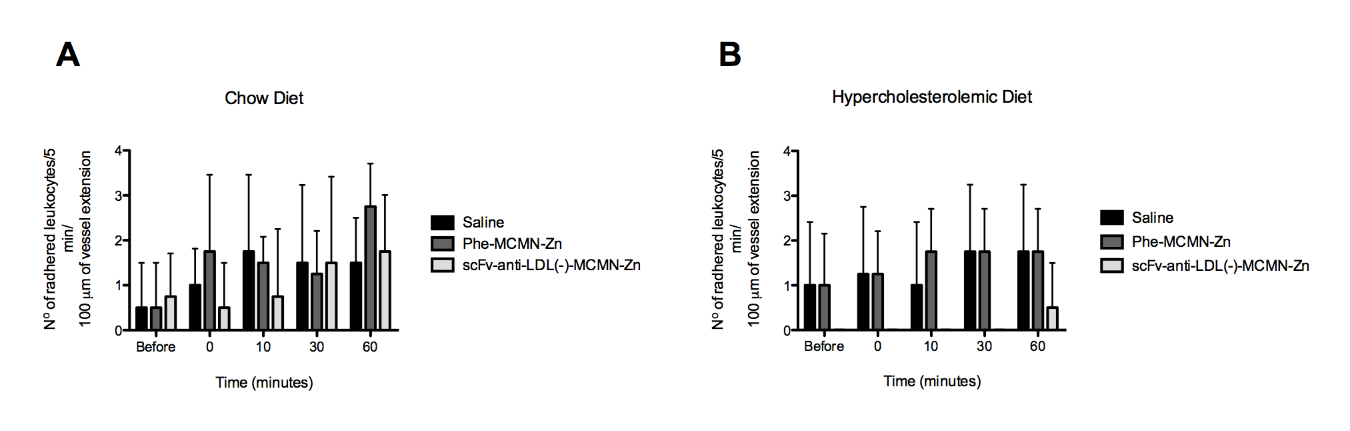


Figure S15. Effects of the intravenous injection of scFv-anti-LDL(-)-MCMN-Zn nanoformulation on the leukocyte-endothelium interactions. 12-week-old male *Ldlr^-/-^* mice previously fed with chow diet (A) or hypercholesterolemic diet (B) for 8 weeks received intravenous injection of 0.9% Saline, 5 x 10^5^ particles/Kg body weight of Phe-MCMN-Zn nanocapsules or 5 x 10^5^ particles/Kg body weight (corresponding to 5 mg scFv/Kg body weight) of scFv-anti-LDL(-)-MCMN-Zn nanoformulation. Number of adhered leukocytes in the microcirculation of the cremaster muscle was assessed by intravital microscopy at baseline, 10, 30 e 60 minutes after the injections. Data are expressed as mean ± SD.


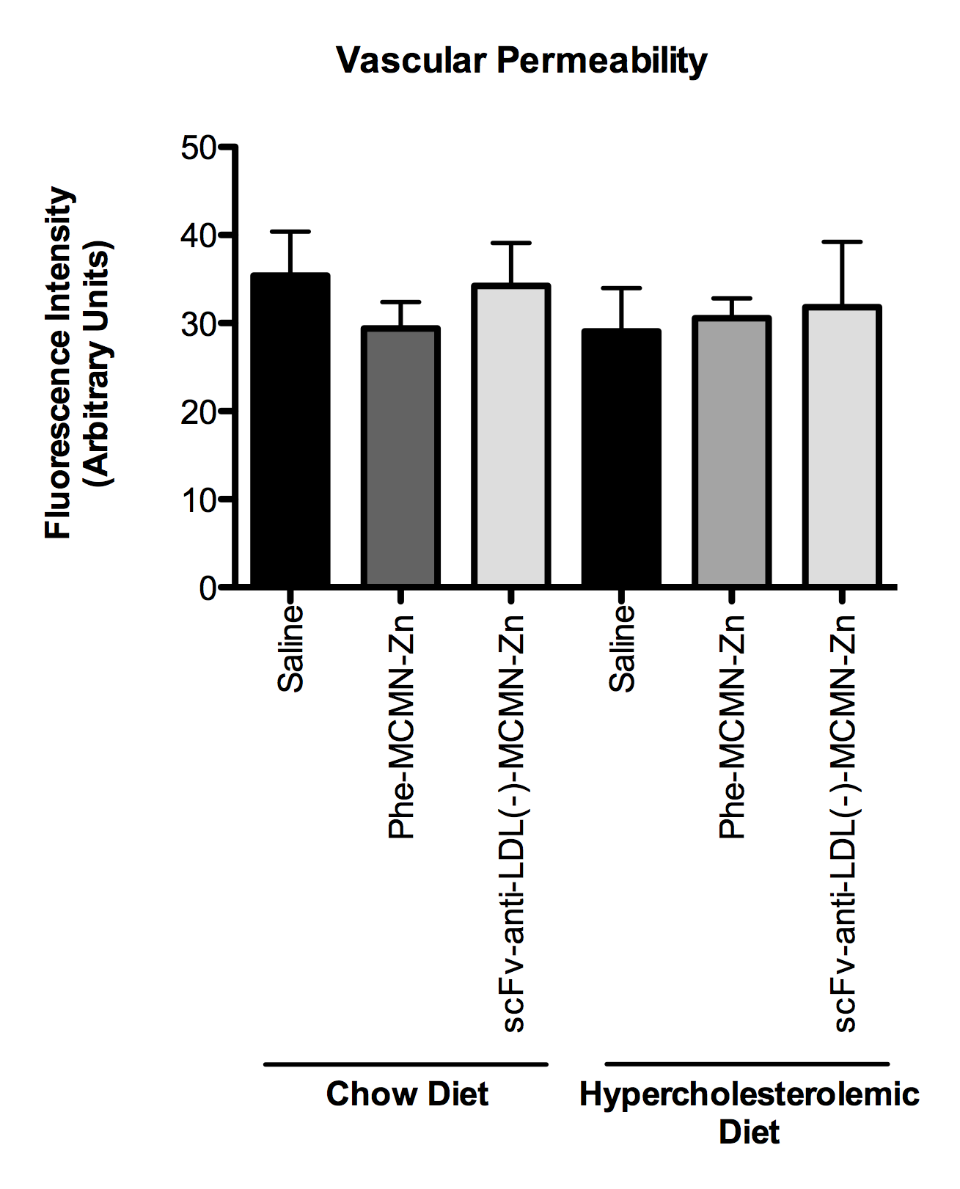


Figure S16. Effects of the intravenous injection of scFv-anti-LDL(-)-MCMN-Zn nanoformulation on vascular permeability. 12-week-old male *Ldlr^-/-^* mice previously fed with chow diet (A) or hypercholesterolemic diet (B) for 8 weeks received intravenous injection of 0.9% Saline, 5 x 10^5^ particles/Kg body weight of Phe-MCMN-Zn nanocapsules or 5 x 10^5^ particles/Kg body weight (corresponding to 5 mg scFv/Kg body weight) of scFv-anti-LDL(-)-MCMN-Zn nanoformulation. Vascular permeability was analyzed 1 hour after a further injection of FITC-albumin (50 mg/kg, 100 µL, Sigma-Aldrich, EUA) with an optical microscope (Axioplan II, Carl-Zeiss, Germany) and a video camera (ZVS, 3C75DE, Carl-Zeiss, Germany). Data are expressed as mean ± SD.

**Tables**

Table S1: Primers used for the quantitative RT-PCR experiments.

| Gene | Forward | Reverse |
| --- | --- | --- |
| *GAPDH^1^* | 5’TGCACCACCAACTGCTTAGC3’ | 5’GGCATGGACTGTGGTCATGAG3’ |
| *IL1B^2^* | 5’GGACAAGCTGAGGAAGATGC3’ | 5’TCGTTATCCCATGTGTCGAA3’ |
| *Il1b^3^* | 5’TTCCCATTAGACAACTGCACTAC3’ | 5’GTCGTTGCTTGGTTCTCCTT3’ |
| *MCP1^4^* | 5’CCCCAGTCACCTGCTGTTAT3’ | 5’TGGAATCCTGAACCCACTTC3’ |
| *Rpl13a^5^* | 5’TCCTCAAGACCAACGGACTCCT3’ | 5’AACCTTTGGTCCCCACTTCCCT3’ |

1. GenBank: CP068262.1; 2. NCBI Reference Sequence: NM_000576.3; 3. NCBI Reference Sequence: NM_008361.4; 4. NCBI Reference Sequence: NM_002982.4; 5. NCBI Reference Sequence: NM_009438.5.

Table S2. Physicochemical characterization of lecithin-lipid-core nanocapsules obtained with different amounts of lecithin: pH, volume-weighted mean diameter (D[4,3]_v_), polydispersity (SPAN), median diameter calculated by number of particles and zeta potential (n=3).

| **Formulation** | **pH** | **D[4,3]_v_ (nm)** | **SPAN** | **d(0.5)_n_ (nm)** | **Zeta potential (mV)** |
| --- | --- | --- | --- | --- | --- |
| LNC_3_ | 6.1 ± 0.6 | 127 ± 2 | 1 ± 0 | 89 ± 1 | -8.2 ± 2.4 |
| LNC_5_ | 5.8 ± 0.4 | 125 ± 3 | 1 ± 0 | 88 ± 2 | -12.3 ± 2.7 |
| LNC_8_ | 6.1 ± 0.4 | 125 ± 1 | 1 ± 0 | 80 ± 7 | -18.0 ± 1.9 |
| LNC_9_ | 6.0 ± 0.2 | 125 ± 4 | 1 ± 0 | 85 ± 1 | -20.0 ± 3.6 |
| LNC_10_ | 4.4 ±0.6 | 2354 ± 1757 | 35 ± 28 | 89 ± 4 | -19.0 ± 7.8 |
| LNC_12_ | 5.9 ± 1.1 | 1468 ± 79 | 10 ± 15 | 86 ± 2 | -20.3 ± 0.7 |
| LNC_14_ | 4.6 ± 0.5 | 9048 ± 9335 | 56 ± 58 | 75 ± 12 | -21.7 ± 4.5 |

Table S3. Physicochemical characterization of chitosan-lecithin-coated lipid-core nanocapsules obtained with different concentrations of chitosan in acetic acid solution: pH, volume-weighted mean diameter (D[4,3]_v_) and zeta potential (n=3).

| Formulation | pH | D[4,3]_V_ (nm) | Zeta potential (mV) |
| --- | --- | --- | --- |
| LNC^+^_0.5_ | 3.6 ± 0.1 | 161 ± 10 | + 9.6 ± 4.4 |
| LNC^+^_0.7_ | 3.6 ± 0.1 | 134 ± 1 | + 15.3 ± 1.3 |
| LNC^+^_0.8_ | 3.7 ± 0.0 | 128 ±5 | +17.6 ± 1.8 |
| LNC^+^_0.9_ | 3.7 ± 0.1 | 131 ± 2 | +16.0 ± 0.2 |
| LNC^+^_1.0_ | 3.8 ± 0.1 | 132 ± 1 | +19.7 ± 2.0 |
| LNC^+^_1.1_ | 3.8 ± 0.1 | 126 ± 7 | +19.6 ± 3.3 |
| LNC^+^_1.2_ | 3.6 ± 0.1 | 132 ± 1 | +19.2 ± 2.4 |
| LNC^+^_1.3_ | 3.9 ± 0.1 | 129 ± 2 | +23.3 ± 2.7 |
| LNC^+^_1.4_ | 4.0 ± 0.1 | 130 ± 3 | +21.9 ± 0.8 |

Table S4. Volume-weighted mean diameter (D[4,3]_v_) of formulations after storage at 5±2 °C (n=3).

| Time (days) | D[4,3]_V_ (nm) | | |
| --- | --- | --- | --- |
|  | LNC^+^_0.5_ | LNC^+^_0.7_ | LNC^+^_1.4_ |
| **1** | 183 ± 17 | 132 ± 1 | 130 ± 2 |
| **7** | 2112 ± 3275 | 135 ± 1 | 618 ± 547 |
| **14** | 724 ± 899 | 134 ± 2 | 1297 ± 1724 |
| **21** | 1726 ± 2764 | 132 ± 2 | 663 ± 448 |

Table S5. Murine blood cell counts after one hour intravenous injection of saline, Phe-MCMN-Zn nanocapsules and scFv-anti-LDL(-)-MCMN-Zn nanoformulation.

|  | Chow diet | | |  | Hypercholesterolemic diet | | |
| --- | --- | --- | --- | --- | --- | --- | --- |
|  | Saline | Phe-MCMN-Zn | scFv-anti-LDL(-)-MCMN-Zn |  | Saline | Phe-MCMN-Zn | scFv-anti-LDL(-)-MCMN-Zn |
| Total Leukocytes (10^6^/mm^3^) | 4.05 ± 0.21 | 4.65 ± 0.46 | 4.4 ± 0.36 |  | 4.8 ± 0.62 | 4.5 ± 0.35 | 4.6 ± 0.54 |
| Neutrophils (%) | 30.7 ± 11 | 32.7 ± 6.45 | 30.75 ± 10.9 |  | 39.5± 1.65 | 35.5 ± 7.53 | 37.2 ± 3.3 |
| Lymphocytes (%) | 59 ± 7.7 | 58.5 ± 5.1 | 60 ± 7.1 |  | 56.25 ± 1.47 | 59.5 ± 5.93 | 57 ± 4.94 |
| Monocytes (%) | 7.5 ± 2.69 | 7.75 ± 2.38 | 4 ± 1.58 |  | 4 ± 1.22 | 5.25 ± 1.47 | 5.5 ± 0.5 |
| Eosinophils (%) | 0.5 ± 0.5 | 1.25 ± 0.89 | 1.25 ± 0.43 |  | 1.25 ± 0.82 | 1.25 ± 0.43 | 1.25 ± 0.82 |

Data are expressed as mean±SD (n=6); *p* > 0.05.

Table S6. Biochemical parameters of the urine of mice after one hour intravenous injection of saline, Phe-MCMN-Zn nanocapsules and scFv-anti-LDL(-)-MCMN-Zn nanoformulation.

|  | Chow diet | | |  | Hypercholesterolemic diet | | |
| --- | --- | --- | --- | --- | --- | --- | --- |
|  | Saline | Phe-MCMN-Zn | scFv-anti-LDL(-)-MCMN-Zn | | Saline | Phe-MCMN-Zn | scFv-anti-LDL(-)-MCMN-Zn |
| **Blood** | Negative | Negative | Negative |  | Negative | Negative | Negative |
| **Urobilinogen** | Normal | Normal | Normal |  | Normal | Normal | Normal |
| **Bilirubin** | Negative | Negative | Negative |  | Negative | Negative | Negative |
| **Protein** | Negative | Negative | Negative |  | Negative | Negative | Negative |
| **Nitrite** | Negative | Negative | Negative |  | Negative | Negative | Negative |
| **Ketone** | Negative | Negative | Negative |  | Negative | Negative | Negative |
| **pH** | 5 | 5 | 5 |  | 5 | 5 | 5 |
| **Density** | 1.03 | 1.03 | 1.03 |  | 1.03 | 1.03 | 1.03 |
| **Leukocytes** | Negative | Negative | Negative |  | Negative | Negative | Negative |

Protein (mg/dL); Ketone (mg/dL); Glucose (mg/dL); Leukocytes (cells/µL). Data are expressed as mean (n=6).
